# LIQA: long-read isoform quantification and analysis

**DOI:** 10.1186/s13059-021-02399-8

**Published:** 2021-06-17

**Authors:** Yu Hu, Li Fang, Xuelian Chen, Jiang F. Zhong, Mingyao Li, Kai Wang

**Affiliations:** 1 Raymond G. Perelman Center for Cellular and Molecular Therapeutics, Children’s Hospital of Philadelphia, Philadelphia, PA 19104 USA; 2 Department of Otolaryngology, Keck School of Medicine, University of Southern California, Los Angeles, CA 90033 USA; 3 Department of Biostatistics, Epidemiology and Informatics, Perelman School of Medicine, University of Pennsylvania, Philadelphia, PA 19104 USA; 4 Department of Pathology and Laboratory Medicine, Perelman School of Medicine, University of Pennsylvania, Philadelphia, PA 19104 USA

## Abstract

Long-read RNA sequencing (RNA-seq) technologies can sequence full-length transcripts, facilitating the exploration of isoform-specific gene expression over short-read RNA-seq. We present LIQA to quantify isoform expression and detect differential alternative splicing (DAS) events using long-read direct mRNA sequencing or cDNA sequencing data. LIQA incorporates base pair quality score and isoform-specific read length information in a survival model to assign different weights across reads, and uses an expectation-maximization algorithm for parameter estimation. We apply LIQA to long-read RNA-seq data from the Universal Human Reference, acute myeloid leukemia, and esophageal squamous epithelial cells and demonstrate its high accuracy in profiling alternative splicing events.

**Supplementary Information:**

The online version contains supplementary material available at 10.1186/s13059-021-02399-8.

## Introduction

RNA splicing is a major mechanism for generating transcriptomic variations, and mis-regulation of splicing is associated with a large array of human diseases caused by hereditary and somatic mutations [1–5]. Over the past decade, RNA sequencing (RNA-seq) has revolutionized transcriptomics studies and facilitated the characterization and understanding of transcriptomic variations in an unbiased fashion. With RNA-seq, we can quantitatively measure isoform-specific gene expression, discover novel and unique transcript isoform signature, and detect differential alternative splicing (DAS) events [6–8]. Until now, short-read RNA-seq has been the method of choice for transcriptomics studies due to its high coverage and single-nucleotide resolution [8]. However, due to limited read length, it is difficult to accurately characterize transcripts using short reads, as 81% of isoforms have length greater than 500 bp in the GENCODE annotation (median = 1543 bp and mean = 2121 bp). This fragmented sequencing of the RNA/cDNA molecules results in biases and has become a barrier for short reads to be correctly mapped to the reference genome, which is crucial for gene or isoform expression estimation and novel or unique isoform detection. As a consequence, transcriptome profiling using short-read RNA-seq is highly biased by read coverage heterogeneity across isoforms. To tackle these challenges, a number of computational tools, including RSEM [9], eXpress [10], TIGAR 2[11], Salmon [12], Sailfish [13], Kallisto [14], Cufflinks [15], CEM [16], PennSeq [17], IsoEM [18], and RD [19], have been developed to quantify isoform expression from short-read RNA-seq data, but different bias correction algorithms can result in conflicting estimates [17]. Overall, quantifying isoform expression using fragmented short reads remains challenging, especially at complex gene loci [20, 21].

In recent years, long-read RNA sequencing has gained popularity due to its potential to overcome the above-mentioned issues when compared to short-read RNA-seq [22, 23]. Previous studies have utilized both single-molecule long-read PacBio Iso-Seq and synthetic long-read MOLECULO methods [24–27]. For Oxford Nanopore sequencing, there are two types of RNA-seq technologies: direct mRNA sequencing and cDNA sequencing. Recently, the Oxford Nanopore Technologies (ONT) MinION has been used to analyze both full-length cDNA samples and mRNA samples derived from tissue cells [28]. Nanopore sequencing is able to generate reads as long as 2 Mbp, which allows a large portion or the entire mRNA or cDNA molecule to be sequenced. Compared to short reads, this advantage of long reads greatly facilitates rare isoform discovery, isoform expression quantification, and DAS event detection.

However, there are still a few unique challenges to analyze long-read RNA-seq data because existing methods developed for Illumina short-read RNA-seq do not have optimal performance when directly used on long-read RNA-seq. This is because parametric bias correction of short-read approaches requires high read coverage and isoform-read assignment is not robust to small range misalignment from long-read data [16, 18, 19, 29]. Methods designed specifically for isoform expression estimation in long-read RNA-seq have only emerged recently. For example, Byrne et al. [30] demonstrated the feasibility of quantifying complex isoform expression using Nanopore RNA-seq data. Tang et al. [31] characterized mutated gene *SF3B1* at isoform level in chronic lymphocytic leukemia cells by leveraging full-length transcript sequencing data generated by Nanopore. While long-read RNA-seq has great potential, the isoform quantification accuracy is still constrained by high error rates and sequencing biases [32], which has yet to be thoroughly accounted for. Specifically, high sequencing error rates (~ 15%) of Nanopore data can result in misalignment of sequencing reads, but current methods assume equal weight for each single-molecule read without accounting for error rate differences when estimating isoform expression. This may complicate isoform usage quantification. In addition, potential read coverage biases are not explicitly taken into account by existing long-read transcriptomic tools [32]. In Nanopore direct mRNA sequencing protocol, pore block and fragmentation can result in truncated reads, leading to biased coverage toward the 3′ end of a transcript [32]. These biases are also shown in data sequenced from cDNA. In the presence of such biases, the accuracy of isoform expression quantification inference can be severely affected, leading to over estimation of expression for isoforms with short length.

**Fig. 1:**
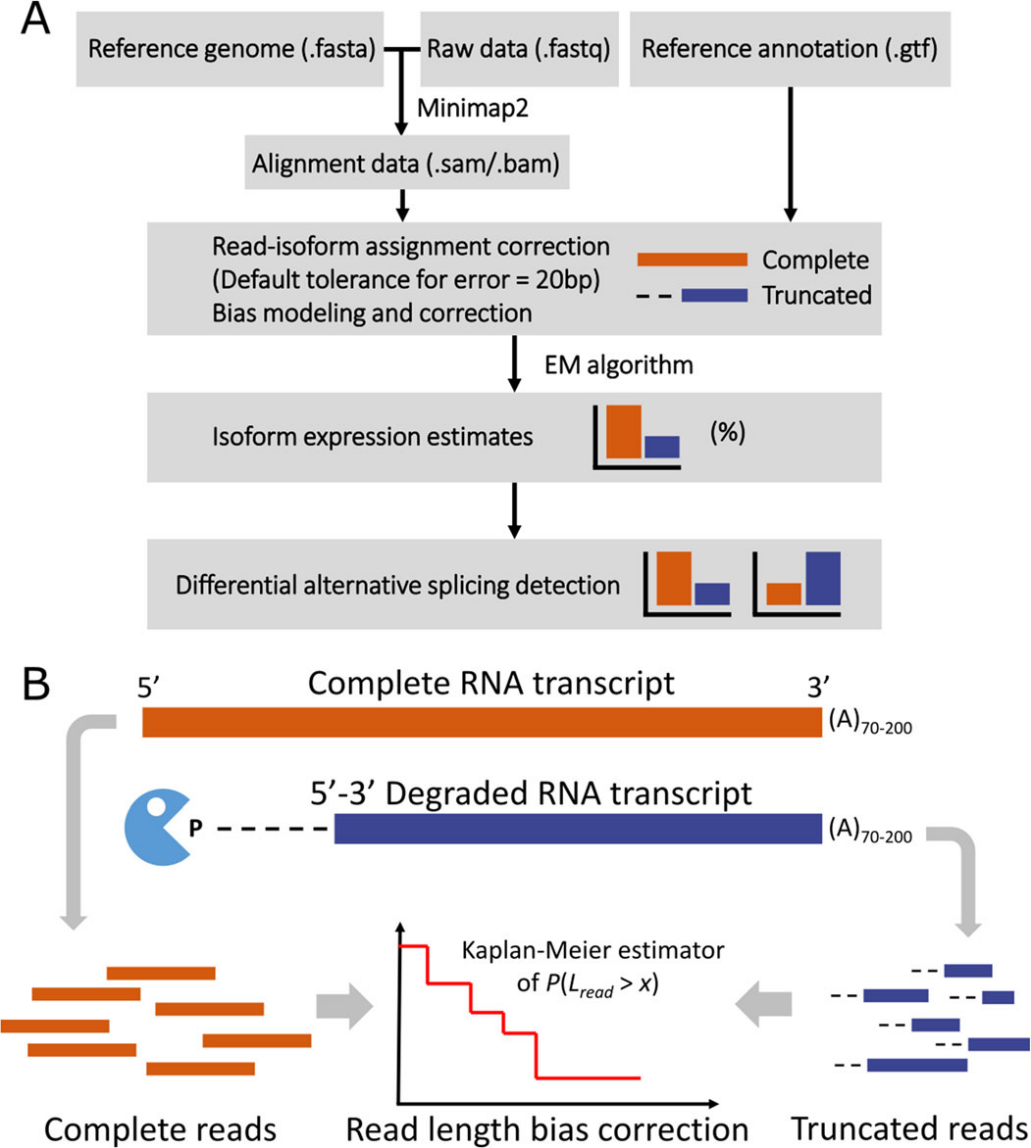
Framework of LIQA. **A** The flowchart to illustrate how LIQA works. The inputs to LIQA are long-read RNA-seq data and isoform annotation file. LIQA models observed splicing information, high error rate of data, and read length bias. The output of LIQA are isoform expression estimates and detected DAS events. **B** Quantification of potential 3′ bias of long-read RNA-seq data. Complete (orange) and truncated (blue) long reads are jointly modeled to correct read length bias by estimating read length distribution

In this article, we present LIQA, a statistical method that allows each read to have its own weight when quantifying isoform expression. Rather than counting single-molecule reads equally, we give a different weight to each read to account for read-specific error rate and alignment bias at the gene (Fig. 1). To evaluate the performance of LIQA, we simulated long data with known ground truth and also sequenced two real samples using Oxford Nanopore sequencing. Our results demonstrate that LIQA is an accurate approach for isoform expression quantification accounting for read coverage bias and high error rate of long-read data.

## Results

### Overview of LIQA

Figure 1 shows the workflow of LIQA and highlights the read length bias correction step. LIQA requires aligned long-read RNA-seq files in BAM or SAM format and isoform annotation file as input. For estimation steps, LIQA first feeds read alignment information to a complete likelihood function and corrects biases for each long read by accounting for quality score and read coverage bias. Second, given that isoform origins are unobserved for some reads, an expectation maximization (EM) algorithm is utilized to achieve the optimal solution of isoform relative abundance estimation. The output values of LIQA are isoform expression estimates. Moreover, LIQA can further detect DAS events given estimated isoform expression values.

To evaluate the performance of LIQA, we compared it with existing long-read based quantification algorithms, including FLAIR [31], Mandalorion [30], TALON [33], and the Oxford Nanopore Pipeline (ONP; https://github.com/nanoporetech/pipeline-transcriptome-de). These methods use long-read RNA-seq data to detect novel isoforms and quantify transcript expression by counting the number of reads, which give equal weight for each read. To make the comparisons fair, we ran LIQA, FLAIR, TALON, Mandalorion, and ONP in quantification mode only with isoform annotation information provided by GENCODE. We benchmarked the performance of each method on both simulated and real data. In addition, we simulated more data to evaluate the performance of LIQA in detecting DAS events between conditions.

### Nanopore RNA-seq data simulation

We conducted a simulation study to evaluate the performance of LIQA and compared it with other state-of-the-art algorithms for isoform expression estimation and DAS detection based on GENCODE v24 annotation. To simulate a realistic dataset with known ground truth, we used NanoSim [34] to generate the ONT RNA-seq data. NanoSim is a fast and scalable read simulator that captures the technology-specific features of ONT data and allows for adjustment upon improvement of Nanopore sequencing technology. This simulator first characterizes Nanopore reads and models features of the library preparation protocols in silico for read simulation. The human genome sequence (GRCh38), transcriptome sequence, and GTF annotation file were downloaded from GENCODE. To make the simulated data close to real studies, we assigned abundance values for each isoform obtained from a real human eye RNA-seq dataset. Using NanoSim, we generated 5 million (5 M) Nanopore reads. To evaluate the impact of sequencing depth on isoform expression quantification, we down-sampled 3 million (3 M), 1 million (1 M), and 0.5 million (0.5 M) reads for the simulated data, respectively. These reads were aligned to the reference genome using minimap2 [35]. Then, we selected genes with 2 or more isoforms (67.2%) to evaluate the performance of LIQA in isoform expression quantification. For each isoform, we compared it with Mandalorion, FLAIR, TALON, and ONP. All methods were run with the same set of simulated aligned data in BAM format as input.

**Fig. 2:**
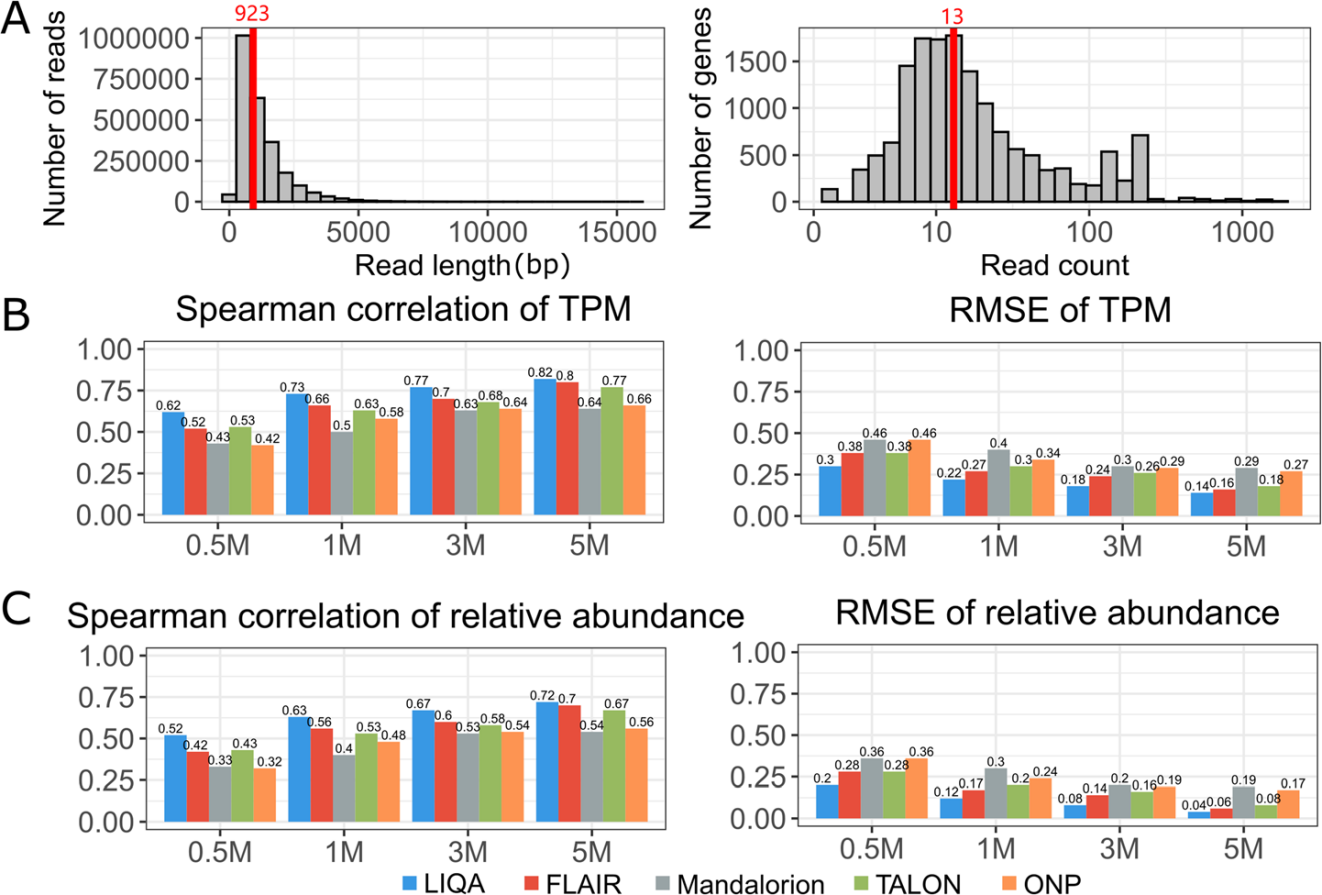
Simulation study results. **A** Characteristics of simulated data with 5 M reads. Read length distribution (left) and read count distribution by genes in log scale (right). **B**,**C** Summary statistics between true and estimated isoform expressions using LIQA (blue), FLAIR (red), Mandalorion (gray), TALON (green), and Oxford Nanopore Pipeline (ONP) (yellow) at different read coverages. **B** Spearman’s correlations (left) and RMSE (right) between estimated and true TPM. **C** Spearman’s correlations (left) and RMSE (right) between estimated and true relative abundance

The characteristics of the simulated data are shown in Fig. 2A and Additional file 1: Fig. S1(A). The median lengths of ONT reads in the 0.5 M, 1 M, 3 M, and 5 M datasets are 896, 922, 1010, and 923 base pairs, respectively. Among the evaluated genes with multiple isoforms (67.2%) based on GENCODE annotation, 13% have two isoforms, 14% have three isoforms, and 73% have four or more isoforms. The simulated isoforms have a wide range of relative abundance (interquartile range = (0.002, 0.75), median = 0.041). In addition, by training the statistical model of NanoSim with a real long-read RNA-seq dataset, the coverage plots of the simulated data capture the features of real ONT RNA-seq data, demonstrating 3′ bias (Additional file 1: Fig. S1(B)). These simulated data thus provide an ideal basis to evaluate the performance of LIQA as the ground truth is known.

### Gene or isoform expression quantification accuracy

For each simulated dataset, we computed a set of measurements to evaluate the estimation accuracy of each method. First, we measured the similarity between the estimated isoform relative abundance and the ground truth by calculating Spearman’s correlation. Second, we measured the estimation accuracy by calculating the root mean squared error (RMSE), defined as $\sqrt{\frac{\sum_g{\sum}_i{\left({\hat{\theta}}_{g,i}-{\theta}_{g,i}\right)}^2}{n}}$, where the summation is taken over all genes and all isoforms within each gene and *n* is the total number of isoforms across all genes. Both statistics were computed at three levels: global gene expression, global isoform expression, and within-gene isoform relative abundances.

**Fig. 3:**
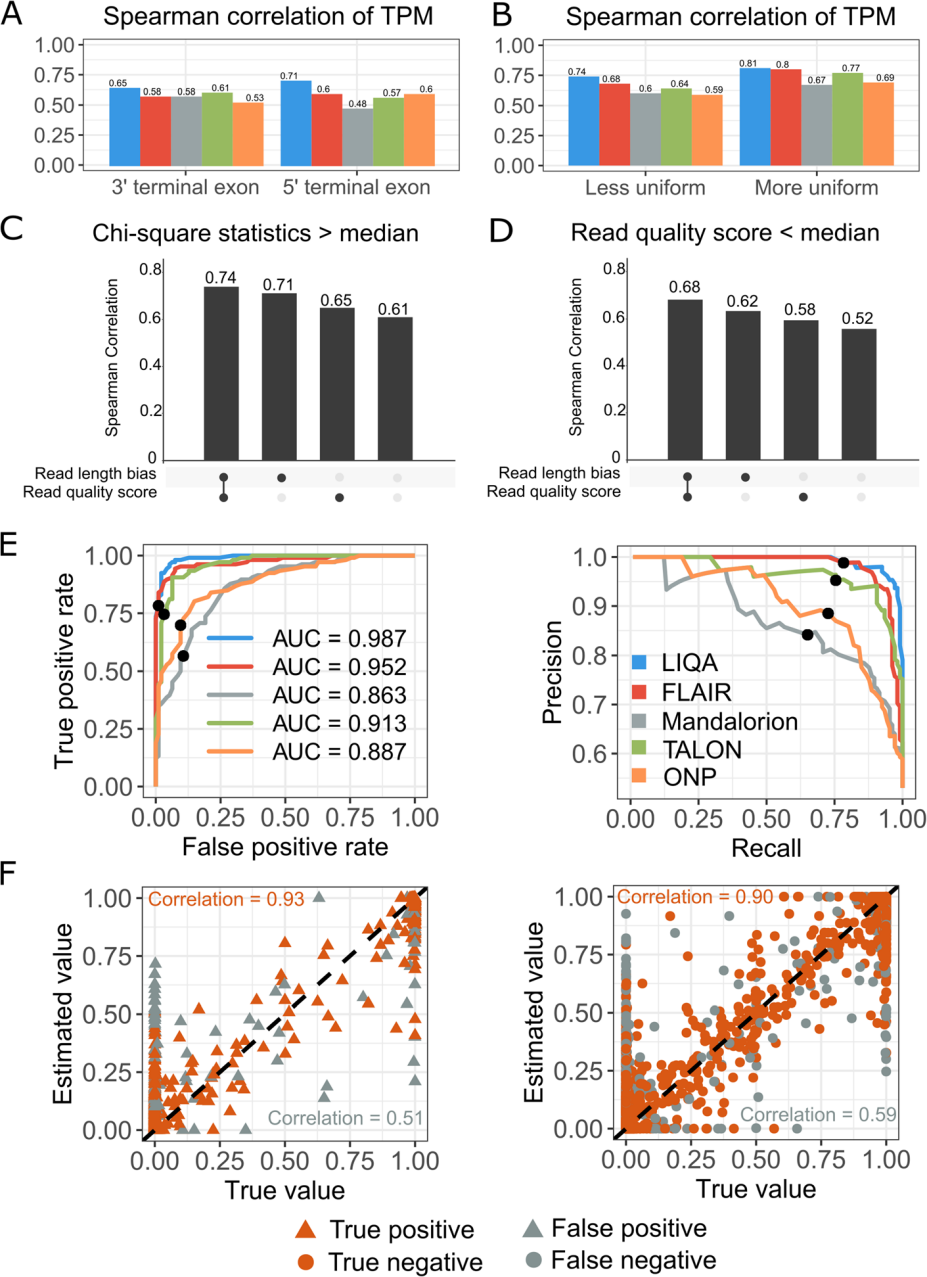
Evaluation of robustness to read length bias and low read quality (**A**–**D**) and comparison of DAS events detection (**E**, **F**) using LIQA (blue), FLAIR (red), Mandalorion (gray), TALON (green), and ONP (yellow). **A** Spearman’s correlations between estimated and true TPM for 3′ and 5′ terminal exons. **B** Spearman’s correlations between estimated and true TPM. Isoforms are stratified by the chi-squared goodness of fit statistic for uniformity. The left panel is for those isoforms in which the read coverage distribution is less uniform, and the right panel is for the remaining isoforms. **C**,**D** Spearman’s correlation comparison between different models (full model, read length model, read quality model, and baseline model) based on isoforms with chi-squared statistic more than the median (**C**) and isoforms with average read quality score less than the median (**D**). **E** ROC curve and precision recall curve of different methods for DAS gene detection. Threshold FDR < 0.05 is highlighted using black dot. **F** Scatter plot of true and estimated isoform relative abundance from the first DAS simulation dataset. LIQA’s prediction and ground truth are marked for each isoform

Figure 2B,C (Additional file 1: Fig. S2) shows the summary statistics between estimated and true values of isoform expression (global isoform expression and isoform relative abundances) at different read coverages. Spearman’s correlation and RMSE were calculated for all five methods. LIQA has higher Spearman’s correlation than other methods for simulated datasets with low sequencing depth (0.5 M) (Additional file 1: Table S2). For simulated data with high sequencing depth (3 M, 5 M), Spearman’s correlation differences between LIQA, FLAIR, and TALON are not significant (Additional file 1: Table S2). Figure 2C gives summary statistics of relative abundance estimates for the five methods. For relative abundance estimation, LIQA outperforms FLAIR and TALON with 6.6% and 7% lower RMSE on average, respectively. Comparison results at gene level reveal a similar pattern (Additional file 1: Fig. S4, Table S5). The improved performance of LIQA is likely due to its use of the EM algorithm, which assigns unequal weight to each read to better account for mapping uncertainty and read mapping bias (Fig. 3C,D and Additional file 1: Table S6, Table S7). In contrast, FLAIR, TALON, and Mandalorion provide discrete estimations by directly counting the number of reads aligned to each corresponding gene or isoform. Due to the limited read coverage of ONT RNA-seq data, it is not surprising that they yield lower estimation accuracy.

To evaluate the robustness of LIQA to a more complex isoform annotation, we analyzed 5 M simulation dataset based on GENCODE v37 annotation. For major use isoforms, as shown in Additional file 1: Fig. S4(D), LIQA still yields 7% higher Spearman correlation of TPM and relative abundance estimates than second best approach FLAIR. For over-annotation, we simulated RNA-seq reads based on 66% of the GENCODE v37 annotation. We then analyzed the simulated data with various methods using 100% of the GENCODE annotation, corresponding to 50% more of the true annotation. Additional file 1: Fig. S4(E) shows the Spearman correlation results of over-annotation. We find that the quantification accuracy of LIQA is nearly unchanged (1% less). For under-annotation, we simulated RNA-seq reads based on 100% of the GENCODE v37 annotation. We then analyzed the simulated data using 50% of the GENCODE annotation, corresponding to 50% less of the true annotation. Additional file 1: Fig. S4(E) shows Spearman’s correlation results of under-annotation. The estimation accuracy is 10% lower when 50% less of the true annotation was used in the analysis.

Next, we evaluated the robustness of LIQA to 3′ read coverage bias (Fig. 3 and Additional file 1: Fig. S3). First, we compared the accuracy statistics for 5′ terminal exon and 3′ terminal exon of each isoform. Isoform expression with non-uniform read coverage is more challenging to estimate because the 5′ end is less likely to be covered by sequencing reads compared to 3′ end. Figure 3A shows the comparison of Spearman’s correlation for five methods with 0.5 M read coverage. LIQA is more accurate than the other four methods at 5′ terminal exon, especially when sequencing depth is low (Additional file 1: Table S4). Spearman’s correlation coefficient of LIQA is 11% higher than the second best performing method FLAIR for 5′ terminal exons, while only 6% higher for 3′ exons. This improved performance of LIQA in terminal exon quantification is also demonstrated by RMSE values. LIQA has 8–15% improvement of RMSE values compared to other methods. Second, we considered the chi-square statistics that measures the goodness of fit of coverage uniformity. Then, we divided the isoforms into two categories based on median of the corresponding measure (chi-square statistic > median, chi-square statistic < median) and summarized Spearman’s correlation coefficient and RMSE for each group of isoforms. For isoforms with more uniform read coverage, Spearman’s correlations of LIQA and FLAIR are close. However, despite reduced Spearman’s correlation value, LIQA is more accurate than the other four methods for isoforms with less uniform read coverage (chi-square statistic > median) (Additional file 1: Table S4). The improvement of LIQA compared to FLAIR is 5% higher for these isoforms. This is likely because LIQA models potential truncated reads which result in 3′ coverage bias when quantifying isoform expression.

Moreover, we assessed the impact of modeling read length bias and read quality score on the accuracy of isoform expression estimation. Figure 3C,D shows the comparison of isoform estimation accuracy using different models. For isoforms with less uniform read coverage (chi-square statistics > median), model with read length bias correction has 9% (full model vs read quality model) and 10% (read length model vs baseline model) higher Spearman’s correlation. For genes with less average read quality, model with read quality score has 6% higher Spearman’s correlation. Overall, isoform estimation accuracy drops noticeably when using baseline model (Additional file 1: Table S7). This comparison demonstrates the advantage of LIQA in handling read length bias and 3′ bias correction over other approaches.

### Differential alternative splicing (DAS) detection

Next, we evaluated the performance of LIQA in DAS detection. More ONT RNA-seq data across multiple samples (*10* cases and *10* controls) were simulated for 10 times. NanoSim generated 3 million reads based on the GENCODE annotation per sample. To make true DAS events more realistic, we sampled relative abundances of isoforms from a Dirichlet distribution with mean and variance parameters estimated from a human eye RNA-seq dataset. Similarly, these simulated data were mapped to the hg38 human reference genome using minimap2. Isoform expression and usage difference between conditions were quantified using LIQA, FLAIR, TALON, Mandalorion, and ONP, respectively. We first compared the performance of DAS detection between these methods using three summary statistics. After FDR control, we measured the recalls (power) of our method by calculating the proportion of correctly predicted DAS events among true DAS events. Second, we obtained precisions by calculating the proportion of correctly predicted DAS events among predicted DAS events. Additionally, F1 score ($\mathrm{F}1\ \mathrm{score}=2\times \frac{\mathrm{precision}\bullet \mathrm{recall}}{\mathrm{precision}+\mathrm{recall}}$) was summarized to average the precision and recall values. As shown in Additional file 1: Fig. S5(B), LIQA, FLAIR, and TALON are more powerful than others for all three evaluation metrics. This is not surprising because Mandalorion and ONP have lower accuracy in isoform expression estimation. For recall value, FLAIR (mean = 0.809, SD = 0.041) gives better and more consistent performance across 10 simulations than LIQA (mean = 0.776, SD = 0.058). However, in terms of precision value, LIQA (mean = 0.915, SD = 0.043) yields less false positives than FLAIR (mean = 0.884, SD = 0.051). LIQA, FLAIR, and TALON had similar performance in detecting DAS events based on F1 score. Furthermore, we generated ROC curve and precision recall curve to compare the performance between methods at different FDR thresholds (Fig. 3E and Additional file 1: Fig. S6, S7). As shown in Fig. 3E, LIQA achieved AUC = 0.94 after FDR control (FDR < 0.05). Given FDR threshold equals to 0.05, LIQA gave the best performance with precision = 0.98 and recall = 0.78. Compared to LIQA, the second best performing method FLAIR yields 0.1%, 0.3%, and 3.5% less in precision, recall, and AUC respectively. In addition, we examined isoform relative abundance estimation accuracy from correct and incorrect detected DAS genes by LIQA (Fig. 3F). After FDR control, we identified that 537 out of 2465 genes are significantly differential spliced, which 431 are true positives and 1836 are true negatives. For these correctly predicted genes, true isoform relative abundance is highly correlated with LIQA’s estimates (Spearman’s correlation = 0.91). For false positive and negative genes, Spearman’s correlation is 38% lower compared to true positive and negative. This is not surprising because accurate estimation of isoform expression level leverages the power of regression model in detecting DAS events.

### Application to the Universal Human Reference (UHR) RNA-seq data

As NanoSim generates ONT RNA-seq data based on trained parametric statistical model, we recognized that simulated data is hardly a full representation of reality. To evaluate the performance of LIQA in a real setting, we sequenced the Universal Human Reference sample with Nanopore Direct mRNA sequencing (Additional file 1: Fig. S12). Then, the resulting ONT-RNA-seq data were analyzed using all five long-read-based methods (LIQA, FLAIR, Mandalorion, TALON, ONP). As quantitative real-time polymerase chain reaction (qRT-PCR) is considered as the most reliable technology for measuring true isoform abundance, we downloaded the qRT-PCR measurements from the MAQC project under Gene Expression Omnibus with accession number GSE5350. As part of the MAQC project, the expression levels of 894 isoforms were measured by TaqMan Gene Expression Assay based qRT-PCR. Additionally, we downloaded the UHR short-read RNA-seq data generated using the Illumina platform. This dataset was analyzed using Cufflinks [15], CEM [16], Salmon [36], and Kallisto [14] to compare the performance in isoform quantification between long reads and short reads. Specifically, we mapped ONT and Illumina sequenced reads to the reference genome using Minimap2 [35] and STAR [37], respectively, and applied each quantification method to the RNA-seq data. qRT-PCR measurements were treated as gold standard to compare the performance across methods. We note that 563 of the 894 transcripts with qRT-PCR measurements are from genes with a single isoform. Estimation results from these genes were served as positive controls (Additional file 1: Fig. S8(A)) because estimating isoform-specific expression for these single-transcript genes is trivial. To compare the performance across different methods, we considered those transcripts that are derived from genes with two or more isoforms.

**Fig. 4:**
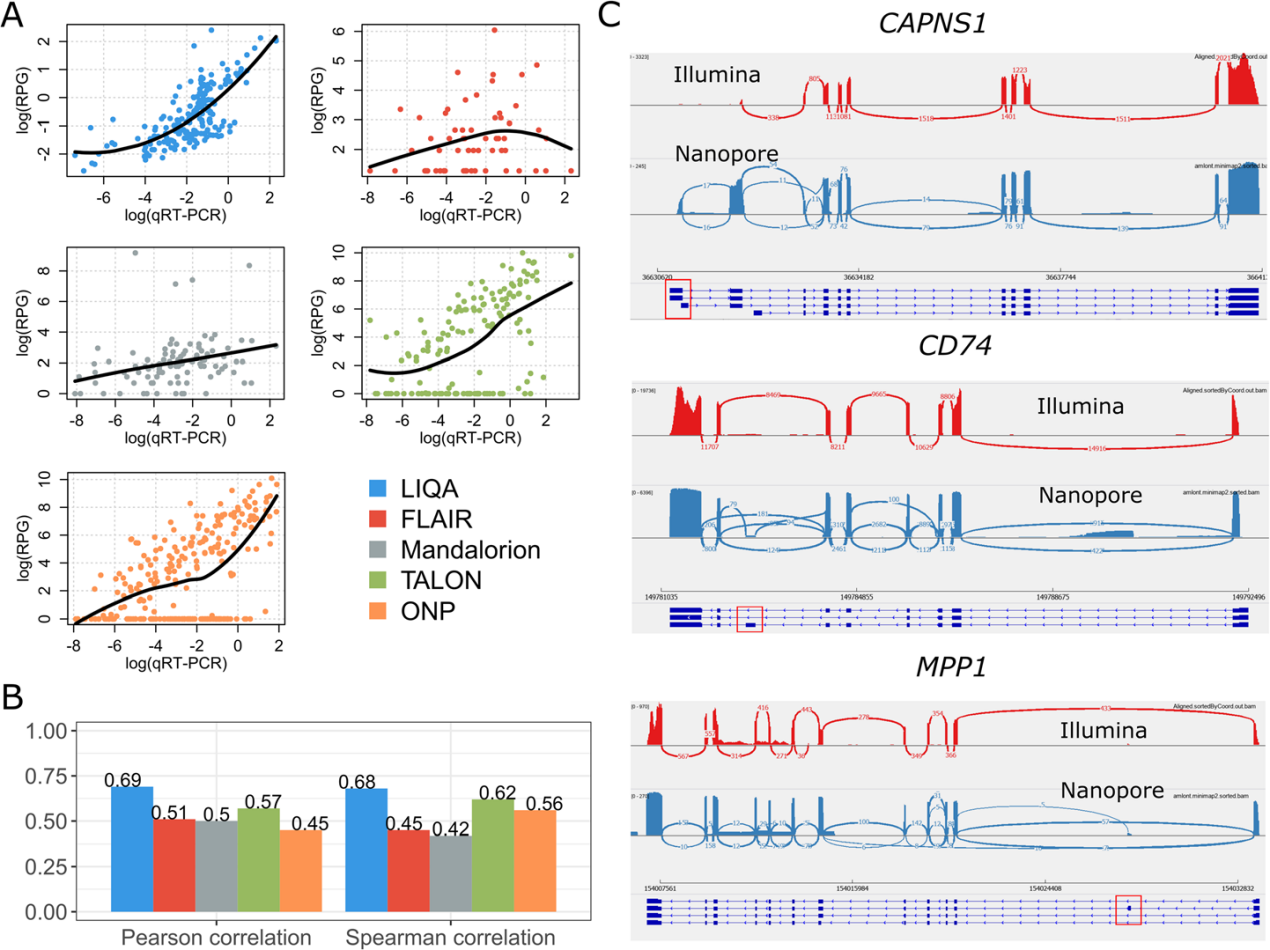
Results on UHR data generated by direct mRNA sequencing on the Nanopore platform. **A** Scatter plots of estimated isoform-specific expression versus qRT-PCR measurements in log scale. Black line represents the local polynomial regression line fitted with estimates and qRT-PCR in log scale. **B** Pearson’s correlation coefficient (left) and Spearman’s correlation coefficient (right) between estimated isoform-specific expression versus qRT-PCR measurements in log scale. **C** Examination of read coverage difference between Illumina and Nanopore data at 3 genes. Informative exonic regions were in red square

To assess the accuracy between estimates and qRT-PCR measurements, we summarized similarity metrics (Spearman’s correlation and Pearson’s correlation) of the isoform abundance values in log scale. As shown in Fig. 4A,B, the estimation accuracy of all methods is lower than simulated data, especially for those transcripts with qRT-PCR measures close to 0. Nevertheless, we observed consistent results in terms of relative performance of different methods with simulation data. LIQA is more accurate than other methods with stronger linear relationship between logarithm estimates and qRT-PCR measurements. However, many of the lowly to moderately expressed isoforms were underestimated using the other methods with their TPM values being compacted toward 0. For ONT data, Spearman’s correlation of LIQA is 0.68, whereas the corresponding values from second best method TALON is 0.62. For Illumina data, Cufflinks seems to correlate with the qRT-PCR measurements better than others (Additional file 1: Fig. S8(B)). The main reason for the better performance of LIQA is likely due to quantifying isoform expression by accounting for isoform length bias and base quality scores. Moreover, we randomly selected 3 genes and generated sashimi plots in Fig. 4C to show the read coverage difference between direct mRNA sequencing and Illumina data. Overall, read distribution of long-read data is less heterogeneous than short-read. Specifically, for gene *CAPNS1*, there is clearly severe 5′ degradation in Illumina data, but with full length and more even coverage across the transcripts for long-read data. Terminal exons at 5′ end in red square are crucial informative regions for splicing analysis, which enable us to differentiate read origin from different isoforms. As shown in Fig. 4C, these exonic regions were captured by Nanopore reads but missed by Illumina reads, which significantly facilitates isoform expression quantification using long-read RNA-seq data. Similarly, sashimi coverage plots of other two genes showed the same pattern, which demonstrates the advantage of long-read data over short-read in alternative splicing study.

Moreover, we conducted additional analysis of another long-read data on UHR with much higher coverage (5.6 million reads), generated on the PacBio sequencing platform [38]. As shown in Additional file 1: Fig. S8(E), Pearson and Spearman’s correlations for each method are generally improved with increasing sequencing depth compared to our Nanopore-based UHR dataset (Fig. 4B). For example, Spearman’s correlation of FLAIR is increased by 0.28 (from 0.45 to 0.73), whereas the corresponding values of LIQA are increased by 0.11. Nevertheless, LIQA still has the best performance among all methods. Based on this real dataset with increasing sequencing depth, we found that LIQA is more robust to low read coverage compared to FLAIR, which performs well when sequencing depth is high. These observations from these two real UHR datasets are consistent with the simulation-based datasets with different sequencing depths (0.5 M, 1 M, 3 M, 5 M).

### Application to Nanopore cDNA sequencing data on a patient with acute myeloid leukemia

AML is a type of blood cancer where abnormal myeloblasts are made by bone marrow [39]. In this study, we sequenced peripheral blood from an acute myeloid leukemia (AML) patient using GridION Nanopore sequencer with Guppy basecalling (https://denbi-nanopore-training-course.readthedocs.io/en/latest/basecalling/basecalling.html). In total, we generated 8,061,683 long reads with 6.6 GB bases (Additional file 1: Fig. S13). We aligned the data against a reference genome (hg38) using minimap2 [35], and 63% long reads (73% bases) are mapped. Then, we analyzed this ONT RNA-seq data with LIQA for genes with at least two isoforms.

**Fig. 5:**
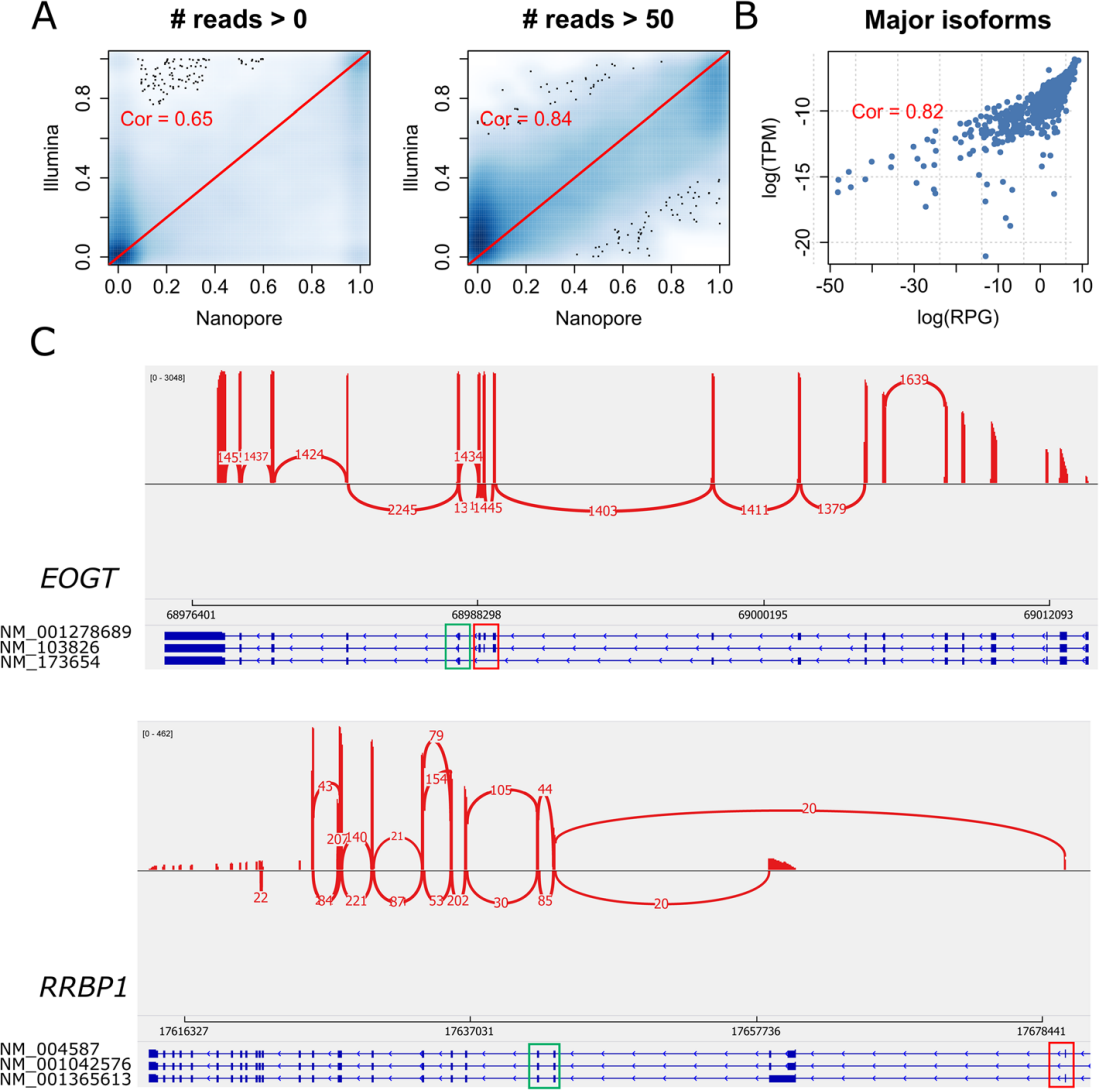
Performance of LIQA using AML data. **A** Scatter plots of estimated isoform relative abundances using long-read data (LIQA) versus short-read data (PennSeq) for all genes (left) and genes with at least 50 read coverage (right). **B** Scatter plot of estimated isoform-specific expression using long-read data (LIQA) versus short-read data (PennSeq) in log scale for all major isoforms. **C** Examination of isoform usage inferred by LIQA. Sashimi plots of gene *EOGT* and *RRBP1*. Informative exonic regions were in green and red squares

We considered two ways to benchmark the performance of LIQA. First, we used PennSeq to analyze short-read sequencing data for the same AML sample and treated the estimates as gold standard. This dataset included 440 M short read with 150 bp in length. Figure 5A shows the scatter plots of isoform relative abundance estimates between long- and short-read data. Spearman’s correlation coefficients were calculated. We found that correlation was improved significantly for genes with at least 50 reads compared to all genes without filtration. Then, we examined the major isoforms (with the highest expression level in a gene) inferred by LIQA. As shown in Fig. 5B, long-read and short-read shared consistent estimates for the major isoforms. This is not surprising because major isoforms were more likely to be sequenced, leading to higher read coverage at unique exonic regions. Second, we visually examined the read coverage plots at unique exonic regions with at least 100 reads to benchmark the performance of LIQA. We generated sashimi plots for two randomly selected genes, *EOGT* and *RRBP1* (Fig. 5C). For gene *EOGT*, the read coverage ratio between exons in red and green squares suggests that isoforms NM_103826 and NM_001278689 expressed much higher than NM_173654. This is consistent with LIQA’ estimates, with relative abundance of NM_173654 less than 0.01. A similar pattern is observed for gene *RRBP1*, where isoform NM_004587 (relative abundance estimates = 0.68) is the major isoform. Results from this AML data demonstrate the robust performance of LIQA to 3′ coverage biases.

### Application to PacBio data on esophageal squamous epithelial cell (ESCC)

Next, we evaluated the performance of LIQA in differential alternative splicing (DAS) detection using an RNA-seq dataset generated from esophageal squamous epithelial cell (ESCC) [40]. This dataset includes PacBio SMRT reads generated from normal immortalized and cancerous esophageal squamous epithelial cell lines. The RNA-seq data were downloaded from Gene Expression Omnibus (PRJNA515570). We applied LIQA to detect differential isoform usage between normal-like and cancer cells. Known splicing differences in existing studies were treated as ground truth to evaluate LIQA’s performance in characterizing isoform usage across samples. In addition, short-read data from these two samples were sequenced using the Illumina platform, which allows us to compare the consistency and accuracy of DAS detection between long-read and short-read data.

**Fig. 6:**
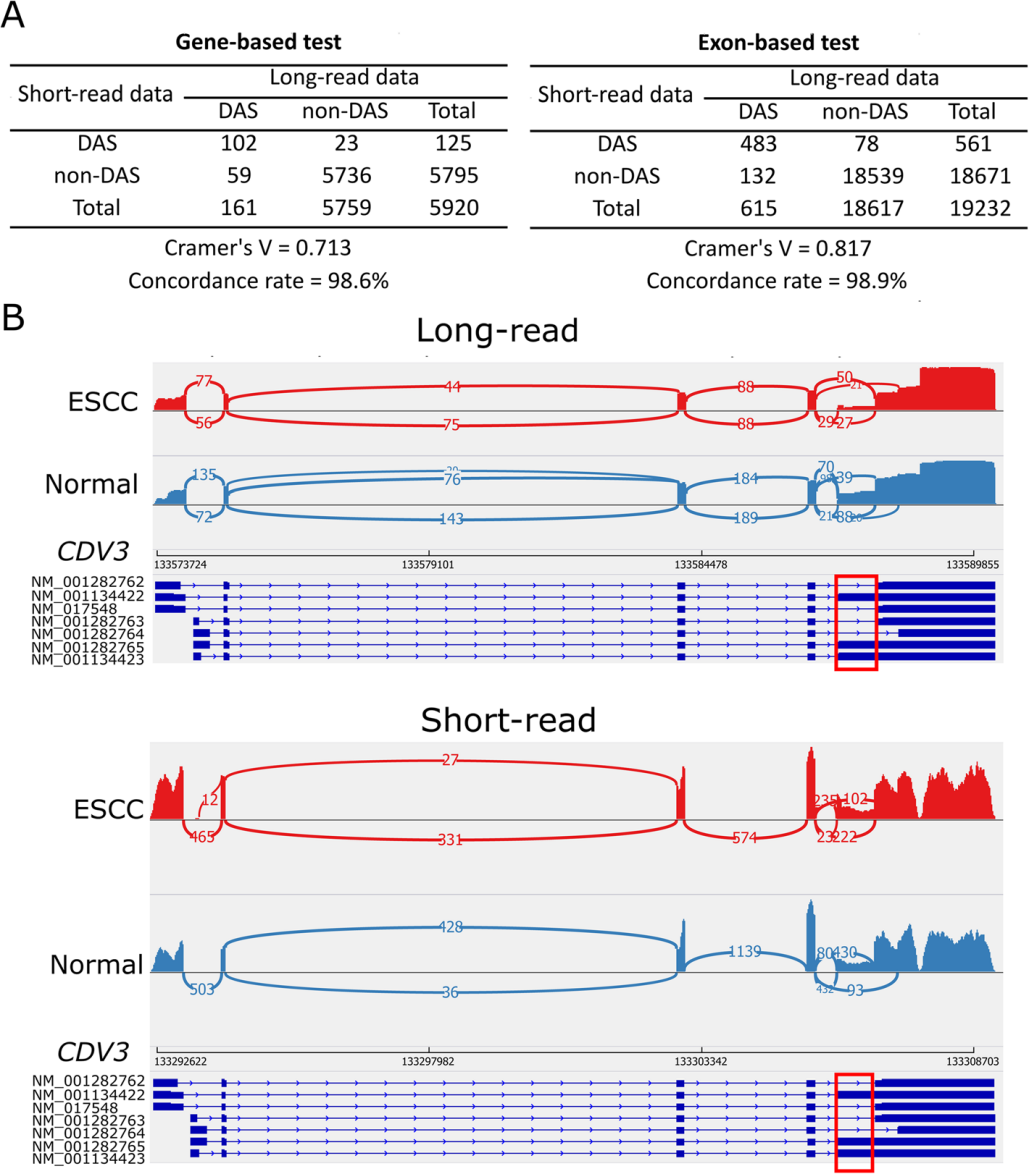
Performance of LIQA using ESCC data. **A** DAS detections between long- and short-read data. Consistency of detected DAS events between long- and short-read data were quantified using Cramer’s V and concordance rate. **B** Examination of AS exon usage inferred by LIQA (long read) but missed by PennDiff (short read). Sashimi plots of gene *CDV3*. Informative exonic regions were red squares

Employing LIQA and PennDiff [41], PacBio, and Illumina data were analyzed to detect DAS events, which are classified into different types, such as skipped exon (SE), alternative 5′ splice site (A5SS), alternative 3′ splice site (A3SS), mutually exclusive exon (MXE), and retained intron (RI). Our results showed that SE is the most frequent type of event among detected DAS between normal-like and cancerous cells, followed by RI, A5SS, and A3SS. MXE is the most infrequent splicing type. As shown in Fig. 6A, detected DAS events by long- and short-read share strong association at both exon and gene level (Cramer’s V > 0.5). Also, the concordance rate between long- and short-read data is greater than 98%. Compared to short-read data, long-read data shows preference in detecting more differential splicing events at both exon and gene level. This is not surprising because read coverage heterogeneity, which might bias DAS detection, is alleviated in long-read data by capturing full-length transcript in each read.

The expression of alternatively spliced isoforms from gene *CDV3* shows difference in cancerous ESCC cells compared to non-cancerous [42, 43]. Figure 6B provides the sashimi plots of a DAS exon at gene *CDV3* detected by LIQA, but was missed by PennDiff using short-read data. From long-read data, it is clear that the relative expression of exon in the red square is lower in cancerous cells than normal-like ESCC. However, this event is missed by short-read data. The read coverage difference between normal-like and cancerous ESCC in sashimi plots indicates the less usage of isoforms (NM_001134422, NM_0011134423, NM_001282765) which include this exon in ESCC cells, suggesting better performance of long-read data.

## Discussion

Accurate estimation of isoform-specific gene expression is a critical step for transcriptome profiling. The emergence of long-read RNA-seq has made it possible to discover complex novel isoforms and quantify isoform usage based on full-length sequenced fragments without amplification bias. However, there are still issues for long-read data, which if not taken into account, can affect the estimations. The major challenges in the analysis of long-read RNA-seq data are the presence of high error rate and potential coverage bias. In this article, we propose LIQA, a statistical method that allows read-specific weight in estimating isoform-specific gene expression. The central idea of our method is to extract error rate information and model non-uniformity read coverage distribution of long-read data. LIQA is the first long-read transcriptomic tool that takes these limitations of long-read RNA-seq data into account. Results of our simulation study and analyses of real data demonstrated that LIQA is more effective in bias correction than the limited existing approaches (Additional file 1: Table S2, S3).

However, we note that there is still room to improve LIQA. LIQA is computationally intensive because the approximation of nonparametric Kaplan-Meier estimator of function *f*(*L*_*r*_) relies on empirical read length distribution and the parameters are estimated using EM algorithm. Based on the analysis of the UHR and AML data, we found that running LIQA is slower than FLAIR and Mandalorion (Additional file 1: Table S1). Currently, we are evaluating the impact of possible parametric functions such as exponential or Weibull distributions for read distribution modeling. This will sacrifice the robustness of isoform expression estimates but the running time can be significantly reduced. We believe it may be worth making this trade-off between computational efficiency and estimation accuracy for LIQA.

We have benchmarked the performance of LIQA with the use of minimap2 for long-read alignment, while there have been several approaches supporting RNA-seq long-read alignment, such as STAR [37], GMAP [44], BLAT [45], BBMap (https://sourceforge.net/projects/bbmap/), and GraphMap 2[46]. LIQA can take SAM or BAM files generated from any listed aligner as input. Nevertheless, we recognize that it is important to evaluate whether LIQA’s superior performance is robust to different aligners. Therefore, we plan to explore more long-read aligner options and settings to benchmark LIQA in the future.

As LIQA is EM algorithm-based, the robustness to parameter initialization is a potential issue. Read-specific weight of LIQA extracts more information from observed data than direct read count strategy as implemented in Mandalorion and FLAIR. Especially, more read coverage is needed for stable approximation of function *f*(*L*_*r*_). For genes with limited reads coverage (less than 5), the likelihood function of LIQA will be flattened, then optimal points are harder to be reached by EM algorithm and estimates may be sensitive to initial values of parameter. Therefore, the sensitivity of LIQA to parameter initialization should be further evaluated and improved.

With full-length transcript sequencing, long-read RNA-seq data (ONT and PacBio) are expected to facilitate transcriptomic studies by offering number of advantages over short reads. For PacBio, HiFi reads are generated with circular consensus sequencing (CCS) using single-molecule consensus, which increases their accuracy over traditional multi-molecule consensus. Compared to Nanopore sequencing, this protocol yields much lower per-base error rate compared to Nanopore sequencing, but potentially shorter reads. Smaller read length may introduce much larger biases in 5′ or 3′ coverage ratio, which requires further adjustment for LIQA to derive more accurate isoform expression estimates. LIQA has custom settings that allow users to flexibly adjust such parameters to handle these platforms. Compared to PacBio (either with traditional library or HiFi library preparation protocols), ONT may be a more promising platform in quantifying isoform expression while generating data with much higher error rate. This is because ONT is currently more affordable with lower per-based cost of data generation, and sequencing data with high read coverage can improve estimation accuracy of isoform usage. For ONT-RNA-seq, there are two types: direct mRNA sequencing and cDNA sequencing. Compared to direct mRNA sequencing, cDNA sequencing allows samples to be barcoded, amplified and requires less amounts of starting materials. Our studies showed that the decrease of read coverage had less impact on LIQA compared to other existing approaches.

In summary, long-read RNA-seq data offer advantages and can help us better understand transcriptomic variations than short-read data. However, better utilizing informative single-molecule sequencing read is not straightforward. LIQA is a robust and effective computational tool to estimate isoform-specific gene expression from long-read RNA-seq data. With the increasing adoption of long-read RNA-seq in biomedical research, we believe LIQA will be well-suited for various transcriptomics studies and offer additional insights beyond gene expression analysis in the future.

## Methods and materials

### Complete likelihood function of LIQA

Given a gene of interest, let ***R*** denote the set of reads that are mapped to the gene of interest, and ***I*** denote the set of known isoforms. For a specific isoform *i* ∈ ***I***, let *θ*_*i*_ denote its relative abundance, with 0 ≤ *θ*_*i*_ ≤ 1 and ∑_*i* ∈ ***I***_*θ*_*i*_ = 1 and *l*_*i*_ denote its length. For each single-molecule long-read *r*, let *L*_*r*_ denote its length. The probability that a read originates from isoform *i* is *P*(iso. = *i*) = *θ*_*i*_. For read-isoform assignment, LIQA accounts for incorrect alignment at splice site. We define parameter ***Z***_***R,I***_ as a ∣***R*** ∣  ×  ∣ ***I***∣, a read-isoform compatibility matrix with ***Z***_***R,I***_(*r*, *i*) = 1 if long-read *r* is generated from a molecule that is originated from isoform *i* (number of mismatch base pairs < 20 bp instead of exact match), and ***Z***_***R,I***_(*r*, *i*) = 0 otherwise. For isoform quantification, our goal is to estimate **Θ** ***=*** {*θ*_***i***_, *i* ***∈ I***} based on RNA-seq long reads mapped to the gene.

With the notation above, the complete data likelihood of the RNA-seq data can be written as

$$ {\displaystyle \begin{array}{l}L\left(\tilde{\varTheta}|\mathbf{R},\mathbf{Z}\right)=\prod \limits_{r\in R}\prod \limits_{r\in I}{\left(P\left(\mathrm{read}=r,\mathrm{read}\;\mathrm{len}.={L}_r,\mathrm{iso}.=i\right)\right)}^{Z_{R,I}\left(r,i\right)}\\ {}=\prod \limits_{r\in R}\prod \limits_{i\in I}{\left(P\left( read=r,\mathrm{read}\;\mathrm{len}.={L}_r|\mathrm{iso}.=i\right)\cdot P\left(\mathrm{iso}.=i\right)\right)}^{Z_{R,I}\left(r,i\right)}\\ {}=\prod \limits_{r\in R}\prod \limits_{i\in I}{\left(P\left( read=r,\mathrm{read}\;\mathrm{len}.={L}_r|\mathrm{iso}.=i\right)\cdot {\theta}_i\right)}^{Z_{R,I}\left(r,i\right)}\end{array}} $$

This formula is based on the fact that given the isoform origin, the probability of observing read alignment can be inferred. The conditional probability of read *r* derived from isoform *i* with length *L*_*r*_ is

$$ P\left(\mathrm{read}=r,\mathrm{read}\ \mathrm{len}.={L}_r\ |\ \mathrm{iso}.=i\right)=q\left(r,i\right)\bullet f\left({L}_r\right|\ \mathrm{iso}.=i\Big) $$

where *q*(*r*, *i*) is isoform-specific read quality score and *f*(*L*_*r*_| iso. = *i*) is isoform-specific read length probability. Essentially, we quantify isoform relative abundance with weighted read assignment. To account for the error-prone manner of Nanopore sequencing data, we consider isoform-specific read quality score $q\left(r,i\right)={\prod}_{j=1}^m{q}_j\left({x}_j,{y}_{(j)}\right)$ where *x* is the sequence of the long-read *r*, *y* is the sequence of the corresponding isoform *i* in the reference genome, and *q*_*j*_(*a*, *b*) is the probability that we observe base *a* at position *j* of the read given that the true base is *b*, which can be calculated as $1-{10}^{-{Q}_j/10}$, with *Q*_*j*_ being the per-based Phred quality score at position *j*.

### Estimation of isoform-specific read length probability *f*(*L*_*r*_| iso. = *i*)

Because read length *L*_*r*_ is not fixed and short prone in Nanopore sequencing, we treat it as a random variable with right skewed distribution density function *f*(∙). Given an isoform, existing long-read methods assume fixed read length for all sequenced reads, and this is equivalent to setting *f*(*L*_*r*_) at 1. However, this assumption does not hold as recent studies suggest that potential 3′ coverage bias exists in long-read RNA-seq data [24, 32, 47]. To offer flexibility in modeling read length distribution, we employ a nonparametric approach. For all long reads mapped to the genome, we categorize them into two groups: complete reads and truncated reads. Accounting for misalignment due to high error rate, the read is treated as complete when the distance between its ending alignment position and any known isoform 5′ end is less than a tolerance threshold (default = 20 bp) (Fig. 1A). This indicates that this read is completely sequenced from a known isoform. Otherwise, the read is considered as truncated. The presence of truncated reads is due to incomplete sequencing or novel isoforms. As known annotated isoforms are treated as gold standard during estimation, we assume true length of truncated read is censored. Given the observed lengths of all complete and truncated reads, we fit them into a survival model, a natural modeling approach for censored data (Additional file 1: Fig. S9, S10, S11). Function $\hat{F}(l)=P\left( read\  len.<l\right)$ can be estimated based on Kaplan-Meier estimator [48], hence we have $f(l)=\hat{F}\left(l+1\right)-\hat{F}(l)$.

Given a gene of interest with ***I =*** {isoform *i* : 1 ≤ *i* ≤ *I*}, isoform-specific read length probability *f*(*L*_*r*_ | iso. = *i*) can be written as

$$ f\left({L}_r\ |\ \mathrm{iso}.=i\right)=\frac{f\left({L}_r\right)\bullet P\left(\mathrm{iso}.=i\ |\ {L}_r\right)}{P\left(\mathrm{iso}.=i\right)}=\frac{f\left({L}_r\right)\bullet {\theta}_i/{\sum}_{l_j>{L}_r}{\theta}_j}{\theta_i}=\frac{f\left({L}_r\right)}{\sum_{l_j>{L}_r}{\theta}_j} $$

This isoform-specific read length probability *f*(*L*_*r*_ | iso. = *i*) captures the sequencing biases due to fragmentation during library preparation or pore-blocking for nanopore data.

### Quantification of isoform expression level

Given that isoform indicators ***Z***_***R,I***_(*r*, *i*) for some reads are not observed from read data, **Θ** are estimated using EM algorithm. Then, we have isoform relative abundance ${\hat{\theta}}_i$. In addition to relative abundance, it is also important to quantify the absolute expression level of an isoform. At gene level, we consider read per gene per 10 K reads (RPG 10 K) as the standard for long-read RNA-seq data. RPG is defined as RPG = *N*/10^4^ where *N* is the number of reads mapped to the gene of interest. With this concept, we estimate the expression level of a particular isoform by replacing *N* with estimated number of long reads originated from isoform *i* (${\mathrm{RPG}}_i=N\bullet {\hat{\theta}}_i/{10}^4$).

### Parameter estimation using the EM algorithm

The complete data likelihood is

$$ L\left(\boldsymbol{\Theta} |\mathbf{R},\mathbf{Z}\right)=\prod \limits_{r\in \boldsymbol{R}}\prod \limits_{i\in \boldsymbol{I}}{\left(q\left(r,i\right)\bullet f\left({L}_r\right)\bullet {\theta}_i\right)}^{{\boldsymbol{Z}}_{\boldsymbol{R},\boldsymbol{I}}\left(r,i\right)} $$

and the update procedure of the EM algorithm is as follows:

**E-step:** We calculate function

$$ {\displaystyle \begin{array}{l}Q\left(\varTheta |{\varTheta}^{(t)}\right)={E}_{Z_{R,I}\mid {\varTheta}^{(t)}}\left[\log\;L\left(\varTheta |R\right)\right]\\ {}=\sum \limits_{r\in R}\sum \limits_{i\in I}{E}_{Z_{R,I}\mid {\varTheta}^{(t)}}\left[{Z}_{R,I}\left(r,i\right)\right]\cdot \log \left(q\left(r,i\right)f\left({L}_r\right){\theta}_i\right)\end{array}} $$

where $$ {E}_{Z_{R,I}\mid {\boldsymbol{\Theta}}^{\left(\boldsymbol{t}\right)}}\left[{\boldsymbol{Z}}_{\boldsymbol{R},\boldsymbol{I}}\left(r,i\right)\right]=\frac{q\left(r,i\right)f\left({L}_r\right){\theta}_i^{(t)}}{\sum \limits_{u\in \boldsymbol{I}}q\left(r,u\right)f\left({L}_r\right){\theta}_u^{(t)}} $$.

**M-step:** We maximize function *Q*(**Θ**| **Θ**^(***t***)^) and have

$$ {\theta}_i^{\left(t+1\right)}=\frac{\sum_{r\in \boldsymbol{R}}{E}_{Z_{R,I}\mid {\boldsymbol{\Theta}}^{\left(\boldsymbol{t}\right)}}\left[{\boldsymbol{Z}}_{\boldsymbol{R},\boldsymbol{I}}\left(r,i\right)\right]}{\mid \boldsymbol{R}\mid } $$

The EM algorithm consists of alternating between the E- and M-steps until convergence. We start the algorithm with **Θ**^(**0**)^ assuming all isoforms are equally expressed and stop when the log likelihood is no longer increasing significantly.

### Detection of differential alternative splicing (DAS) with LIQA

The relative abundance of an isoform takes values between 0 and 1. Therefore, we assume it follows a beta distribution, which is well known as a flexible distribution in modeling proportion because its density can have different shapes depending on the values of the two parameters that characterize the distribution, i.e., *θ*_*i*_~Beta(*μ*_*i*_, *ϕ*_*i*_). The expected value and variance of *θ*_*i*_ are

$$ {\displaystyle \begin{array}{c}E\left({\theta}_i\right)={\mu}_i\\ {} Var\left({\theta}_i\right)=\frac{\mu_i\left(1-{\mu}_i\right)}{1+{\phi}_i}\end{array}} $$

To detect splicing difference of isoform *i* between two groups of samples, we utilized beta regression model with *ϕ*_*i*_ as precision parameter. We apply logit link function and have the model

$$ \mathrm{logit}\left({\theta}_i\right)={\beta}_0+{\beta}_1Z $$

where *Z* is the condition indicator (1 for case; 0 for control), *β*_0_ and *β*_1_ are coefficient parameters.

Since the isoform relative abundances of isoforms within the same gene are correlated, a robust and flexible model is needed when comparing them between conditions at a gene level. To account for this, we utilize Gaussian copula regression model to test splicing difference significance between conditions of correlated isoform relative abundances. The separation of marginal distributions and correlation structure makes Gaussian copula regression versatile in modeling non-normal dependent observations. Therefore, the joint distribution of isoform relative abundances from the same gene is given by

$$ {\Phi}_{I-1}\left({\Phi}^{-1}\left(F\left({\theta}_1|{\beta}_0,{\beta}_1,{\varphi}_1\right)\right),\dots, {\Phi}^{-1}\left(F\left({\theta}_{I-1}|{\beta}_0,{\beta}_{I-1},{\varphi}_{I-1}\right)\right)|\boldsymbol{\varGamma} \right) $$

where *φ*_*i*_ is the dispersion parameter of the marginal generalized linear model for isoform *i*. Φ_*I* − 1_(| ***Γ***) is the cumulative distribution function of multivariate normal random variables with *I* − 1 dimensions and correlation matrix ***Γ*.** We choose to use exchangeable correlation structure for ***Γ*.** Given regression models above, we can detect DAS both for at the isoform level and at gene level. For isoform *i*, we test *H*_0_ : *β*_1_ = 0 vs *H*_1_ : *β*_1_ ≠ 0 to determine splicing change between conditions. For gene *g*, we test ${H}_0:{\beta}_1^1=\dots ={\beta}_1^{I-1}=0\ \mathrm{vs}\ {H}_1:{\beta}_1^i\ne 0$ for any 1 ≤ *i* ≤ *I* − 1.

### Nanopore direct mRNA sequencing of Universal Human Reference RNA-seq data

Universal human reference (UHR) RNA comprises of mixed RNA molecules by a diverse set of 10 cancer cell lines with equal quantities of DNase-treated RNA from adenocarcinoma in mammary gland, hepatoblastoma in liver, adenocarcinoma in cervix, embryonal carcinoma in testis, glioblastoma in brain, melanoma, liposarcoma, histocytic lymphoma in histocyte macrophage, lymphoblastic leukemia, and plasmacytoma in B lymphocyte. This reference sample from MicroArray Quality Control (MAQC) [49–51] project has been utilized in many studies. For example, Gao et al. [52] sequenced this UHR RNA sample and treated it as reference to measure the technical variations of scRNA-seq data. Also, the qRT-PCR measurements of gene or isoform expressions from this sample were used to benchmark and optimize computational tools [17, 53–56]. In this study, we used GridION Nanopore technique to sequence mRNA directly and used Guppy for base calling. In total, we generated 476,000 long reads with 557 MB bases. We aligned the UHR RNA-seq data against a reference genome (hg38) using minimap2 [35], and 95% long reads (89% of total bases) are mapped, demonstrating very high sequencing and basecalling quality. qRT-PCR measurements were downloaded and treated as ground truth to compare the performance between LIQA, FLAIR, Mandalorion, CEM, Cufflinks, and RD.

### Chi-squared goodness of fit statistics of read coverage uniformity

Given an isoform of interest, let *l* denote the length and *O*_*i*_ denote observed read coverage count at base pair position *i*. Total sequencing depth of this isoform $S=\sum \limits_{1\le i\le l}{O}_i$. Under the uniform read coverage assumption, the expected read coverage count at each base pair position *E*_*i*_ = *S*/*l*. We apply chi-squared goodness of fit statistics to measure the difference between observed read coverage and uniform read distribution. The test statistics is

$$ {\chi}^2=\sum \limits_{1\le i\le l}\frac{{\left({O}_i-{E}_i\right)}^2}{E_i} $$

The degree of freedom is isoform length *l*_*i*_ − 1. The higher value of *χ*^2^ indicates that observed read coverage deviates more from uniform read distribution. We calculated *χ*^2^ for each isoform, then divided them into two categories based on median of the corresponding measure (less uniform: *χ*^2^ > median, more uniform *χ*^2^ < median) to evaluate the impact of read coverage distribution on isoform expression quantification.

### Statistical test to compare performance of different methods

We simulated ONT RNA-seq data 20 times to assess the statistical significance when comparing the performance of different methods. Each dataset includes 5 million (5 M) reads. We also down-sampled 3 million (3 M), 1 million (1 M), and 0.5 million (0.5 M) reads for the simulated data to evaluate the impact of sequencing depth on performance improvement of LIQA. We ran all methods with the same set of simulated aligned data in BAM format as input and calculated Spearman’s correlation of TPM and relative abundance between true and estimated values. Based on this metric from 20 simulated datasets, we conducted pairwise comparison of performance difference between all methods using paired Z-test. Mean difference, standard deviations, test statistics, and *P* values were calculated. Moreover, we conducted likelihood ratio test to compare different models of LIQA (full model, read length model, read quality model). Likelihood ratio test statistic Q =  − 2(log*L*_*B*_ − log *L*_*A*_), where *L* is the optimized likelihood function based on different models.

### Availability of data and materials

LIQA is freely available at https://github.com/WGLab/LIQA under GPLv3 license [57]. The direct mRNA sequencing data on UHR has been deposited and available at Gene Expression Omnibus (PRJNA639366) [58]. The cDNA sequencing data on a patient with cancer has been deposited and available at Gene Expression Omnibus (PRJNA640456) [59]. The simulation data used in our study can be reproduced using code provided in the LIQA software repository and NanoSim version 2.0.0. The esophageal squamous epithelial cell PacBio RNA-seq data we applied is from Gene Expression Omnibus (PRJNA515570) [40]. Source code used in the manuscript is available via Zenodo with DOI 10.5281/zenodo.4795477 [60].

## Supplementary Information

**Additional file 1.** Figures S1-S13 and Tables S1-S7.

**Additional file 2.** Review history.

## References

[1] Han J, Xiong J, Wang D, Fu XD (2011). Pre-mRNA splicing: where and when in the nucleus. Trends Cell Biol.

[2] Scotti MM, Swanson MS (2016). RNA mis-splicing in disease. Nat Rev Genet.

[3] Montes M, Sanford BL, Comiskey DF, Chandler DS (2019). RNA splicing and disease: animal models to therapies. Trends Genet.

[4] Li YI, van de Geijn B, Raj A, Knowles DA, Petti AA, Golan D, Gilad Y, Pritchard JK (2016). RNA splicing is a primary link between genetic variation and disease. Science.

[5] Kim HK, Pham MHC, Ko KS, Rhee BD, Han J (2018). Alternative splicing isoforms in health and disease. Pflugers Arch.

[6] Pan Q, Shai O, Lee LJ, Frey BJ, Blencowe BJ (2008). Deep surveying of alternative splicing complexity in the human transcriptome by high-throughput sequencing. Nat Genet.

[7] Wang ET, Sandberg R, Luo S, Khrebtukova I, Zhang L, Mayr C, Kingsmore SF, Schroth GP, Burge CB (2008). Alternative isoform regulation in human tissue transcriptomes. Nature.

[8] Wang Z, Gerstein M, Snyder M (2009). RNA-Seq: a revolutionary tool for transcriptomics. Nat Rev Genet.

[9] Li B, Dewey CN (2011). RSEM: accurate transcript quantification from RNA-Seq data with or without a reference genome. BMC Bioinformatics.

[10] Roberts A, Pachter L (2013). Streaming fragment assignment for real-time analysis of sequencing experiments. Nat Methods.

[11] Nariai N, Kojima K, Mimori T, Sato Y, Kawai Y, Yamaguchi-Kabata Y, Nagasaki M (2014). TIGAR2: sensitive and accurate estimation of transcript isoform expression with longer RNA-Seq reads. BMC Genomics.

[12] Zhang C, Zhang B, Lin LL, Zhao S (2017). Evaluation and comparison of computational tools for RNA-seq isoform quantification. BMC Genomics.

[13] Patro R, Mount SM, Kingsford C (2014). Sailfish enables alignment-free isoform quantification from RNA-seq reads using lightweight algorithms. Nat Biotechnol.

[14] Bray NL, Pimentel H, Melsted P, Pachter L (2016). Near-optimal probabilistic RNA-seq quantification. Nat Biotechnol.

[15] Trapnell C, Roberts A, Goff L, Pertea G, Kim D, Kelley DR, Pimentel H, Salzberg SL, Rinn JL, Pachter L (2012). Differential gene and transcript expression analysis of RNA-seq experiments with TopHat and Cufflinks. Nat Protoc.

[16] Li W, Jiang T (2012). Transcriptome assembly and isoform expression level estimation from biased RNA-Seq reads. Bioinformatics.

[17] Hu Y, Liu Y, Mao X, Jia C, Ferguson JF, Xue C, Reilly MP, Li H, Li M (2014). PennSeq: accurate isoform-specific gene expression quantification in RNA-Seq by modeling non-uniform read distribution. Nucleic Acids Res.

[18] Nicolae M, Mangul S, Mandoiu II, Zelikovsky A (2011). Estimation of alternative splicing isoform frequencies from RNA-Seq data. Algorithms Mol Biol.

[19] Wan L, Yan X, Chen T, Sun F (2012). Modeling RNA degradation for RNA-Seq with applications. Biostatistics.

[20] Steijger T, Abril JF, Engstrom PG, Kokocinski F, Consortium R, Hubbard TJ, Guigo R, Harrow J, Bertone P (2013). Assessment of transcript reconstruction methods for RNA-seq. Nat Methods.

[21] Tilgner H, Grubert F, Sharon D, Snyder MP (2014). Defining a personal, allele-specific, and single-molecule long-read transcriptome. Proc Natl Acad Sci U S A.

[22] Burgess DJ (2018). Genomics: Next regeneration sequencing for reference genomes. Nat Rev Genet.

[23] Pollard MO, Gurdasani D, Mentzer AJ, Porter T, Sandhu MS (2018). Long reads: their purpose and place. Hum Mol Genet.

[24] Sharon D, Tilgner H, Grubert F, Snyder M (2013). A single-molecule long-read survey of the human transcriptome. Nat Biotechnol.

[25] Tilgner H, Jahanbani F, Blauwkamp T, Moshrefi A, Jaeger E, Chen F, Harel I, Bustamante CD, Rasmussen M, Snyder MP (2015). Comprehensive transcriptome analysis using synthetic long-read sequencing reveals molecular co-association of distant splicing events. Nat Biotechnol.

[26] Treutlein B, Gokce O, Quake SR, Sudhof TC (2014). Cartography of neurexin alternative splicing mapped by single-molecule long-read mRNA sequencing. Proc Natl Acad Sci U S A.

[27] Vollmers C, Penland L, Kanbar JN, Quake SR (2015). Novel exons and splice variants in the human antibody heavy chain identified by single cell and single molecule sequencing. PLoS One.

[28] Oikonomopoulos S, Wang YC, Djambazian H, Badescu D, Ragoussis J (2016). Benchmarking of the Oxford Nanopore MinION sequencing for quantitative and qualitative assessment of cDNA populations. Sci Rep.

[29] Roberts A, Trapnell C, Donaghey J, Rinn JL, Pachter L (2011). Improving RNA-Seq expression estimates by correcting for fragment bias. Genome Biol.

[30] Byrne A, Beaudin AE, Olsen HE, Jain M, Cole C, Palmer T, DuBois RM, Forsberg EC, Akeson M, Vollmers C (2017). Nanopore long-read RNAseq reveals widespread transcriptional variation among the surface receptors of individual B cells. Nat Commun.

[31] Tang AD, Soulette CM, van Baren MJ, Hart K, Hrabeta-Robinson E, Wu CJ, Brooks AN (2020). Full-length transcript characterization of SF3B1 mutation in chronic lymphocytic leukemia reveals downregulation of retained introns. Nat Commun.

[32] Amarasinghe SL, Su S, Dong X, Zappia L, Ritchie ME, Gouil Q (2020). Opportunities and challenges in long-read sequencing data analysis. Genome Biol.

[33] Wyman D, Balderrama-Gutierrez G, Reese F, Jiang S, Rahmanian S, Forner S, et al. A technology-agnostic long-read analysis pipeline for transcriptome discovery and quantification. BioRxiv. 2020. 10.1101/672931

[34] Hafezqorani S, Yang C, Lo T, Nip KM, Warren RL, Birol I. Trans-NanoSim characterizes and simulates nanopore RNA-sequencing data. Gigascience. 2020;9:giaa061. 10.1093/gigascience/giaa061 10.1093/gigascience/giaa061 PMC7285873 32520350

[35] Li H (2018). Minimap2: pairwise alignment for nucleotide sequences. Bioinformatics.

[36] Patro R, Duggal G, Love MI, Irizarry RA, Kingsford C (2017). Salmon provides fast and bias-aware quantification of transcript expression. Nat Methods.

[37] Dobin A, Davis CA, Schlesinger F, Drenkow J, Zaleski C, Jha S, Batut P, Chaisson M, Gingeras TR (2013). STAR: ultrafast universal RNA-seq aligner. Bioinformatics.

[38] Sequel II system data release: universal human reference (UHR) iso seq. https://github.com/PacificBiosciences/DevNet/wiki/Sequel-II-System-Data-Release:-Universal-Human-Reference-(UHR)-Iso-Seq.

[39] De Kouchkovsky I, Abdul-Hay M (2016). Acute myeloid leukemia: a comprehensive review and 2016 update. Blood Cancer J.

[40] Cheng YW, Chen YM, Zhao QQ, Zhao X, Wu YR, Chen DZ, Liao LD, Chen Y, Yang Q, Xu LY (2019). Long read single-molecule real-time sequencing elucidates transcriptome-wide heterogeneity and complexity in esophageal squamous cells. Front Genet.

[41] Hu Y, Lin J, Hu J, Hu G, Wang K, Zhang H, Reilly MP, Li M (2018). PennDiff: detecting differential alternative splicing and transcription by RNA sequencing. Bioinformatics.

[42] Xie ZC, Wu HY, Ma FC, Dang YW, Peng ZG, Zhou HF, Chen G (2019). Prognostic alternative splicing signatures and underlying regulatory network in esophageal carcinoma. Am J Transl Res.

[43] Ueno N, Shimizu A, Kanai M, Iwaya Y, Ueda S, Nakayama J, Seo MK (2016). Enhanced expression of fibroblast growth factor receptor 3 IIIc promotes human esophageal carcinoma cell proliferation. J Histochem Cytochem.

[44] Wu TD, Watanabe CK (2005). GMAP: a genomic mapping and alignment program for mRNA and EST sequences. Bioinformatics.

[45] Kent WJ (2002). BLAT--the BLAST-like alignment tool. Genome Res.

[46] Maric J, Sovic I, Krizanovic K, Nagarajan N, Sikic M. Graphmap2-splice-aware RNA-seq mapper for long reads. bioRxiv. 2019. 10.1101/720458.

[47] Kellner S, Burhenne J, Helm M (2010). Detection of RNA modifications. RNA Biol.

[48] Kaplan EL, Meier P (1958). Nonparametric estimation from incomplete observations. J Am Stat Assoc.

[49] Consortium M, Shi L, Reid LH, Jones WD, Shippy R, Warrington JA, Baker SC, Collins PJ, de Longueville F, Kawasaki ES (2006). The MicroArray Quality Control (MAQC) project shows inter- and intraplatform reproducibility of gene expression measurements. Nat Biotechnol.

[50] Shi L, Campbell G, Jones WD, Campagne F, Wen Z, Walker SJ, Su Z, Chu TM, Goodsaid FM, Pusztai L (2010). The MicroArray Quality Control (MAQC)-II study of common practices for the development and validation of microarray-based predictive models. Nat Biotechnol.

[51] Sun P, Sehouli J, Denkert C, Mustea A, Könsgen D, Koch I, Wei L, Lichtenegger W (2005). Expression of estrogen receptor-related receptors, a subfamily of orphan nuclear receptors, as new tumor biomarkers in ovarian cancer cells. J Mol Med.

[52] Gao F, Kim JM, Kim J, Lin M-Y, Liu CY, Russin JJ, Walker CP, Dominguez R, Camarena A, Nguyen JD (2018). Evaluation of biological and technical variations in low-input RNA-Seq and single-cell RNA-Seq. Int J Comput Biol Drug Des.

[53] Xu J, Su Z, Hong H, Thierry-Mieg J, Thierry-Mieg D, Kreil DP, Mason CE, Tong W, Shi L (2014). Cross-platform ultradeep transcriptomic profiling of human reference RNA samples by RNA-Seq. Sci Data.

[54] Garcia-Alonso L, Holland CH, Ibrahim MM, Turei D, Saez-Rodriguez J (2019). Benchmark and integration of resources for the estimation of human transcription factor activities. Genome Res.

[55] Teng M, Love MI, Davis CA, Djebali S, Dobin A, Graveley BR, Li S, Mason CE, Olson S, Pervouchine D (2016). A benchmark for RNA-seq quantification pipelines. Genome Biol.

[56] Hayer KE, Pizarro A, Lahens NF, Hogenesch JB, Grant GR (2015). Benchmark analysis of algorithms for determining and quantifying full-length mRNA splice forms from RNA-seq data. Bioinformatics.

[57] Hu Y, Li M, Wang K. LIQA: long-read isoform quantification and analysis. Github. 2021; https://github.com/WGLab/LIQA 10.1186/s13059-021-02399-8 PMC8212471 34140043

[58] Hu Y, Fang L, Chen X, Zhong JF, Li M, Wang K. Long-read sequencing of reference RNA samples. Datasets Gene Expression Omnibus. 2021. https://www.ncbi.nlm.nih.gov/bioproject/?term=PRJNA639366.

[59] Hu Y, Fang L, Chen X, Zhong JF, Li M, Wang K. Oxford nanopore sequencing of acute myeloid leukemia samples. Datasets Gene Expression Omnibus. https://www.ncbi.nlm.nih.gov/bioproject/?term=PRJNA640456.

[60] Hu Y, Li M, Wang K. LIQA: long-read isoform quantification and analysis. Zenodo. 2021; 10.5281/zenodo.4795477. 10.1186/s13059-021-02399-8 PMC8212471 34140043

