# Supplementary material for: LIQA: long-read isoform quantification and analysis
Source: Genome Biol. 2021 Jun 17;22:182. doi: 10.1186/s13059-021-02399-8 (PMC8212471; doi:10.1186/s13059-021-02399-8)
Supplement: Supplementary file 2 — Additional file 2. Review history. [file 13059_2021_2399_MOESM2_ESM.docx]

Review History

**First round of review**

**Reviewer 1**

**Are you able to assess all statistics in the manuscript, including the appropriateness of statistical tests used?** Yes

**Comments to author:**

The authors present an isoform expression quantification method that performs weighted quantification using long reads with an EM algorithm. They demonstrate good performance in isoform quantification and differential splicing analysis with simulated datasets and they further evaluated quantification with real long read data.

This paper touches on an important and interesting topic to propose a novel quantification method for isoform expression with long read RNA-Seq. The authors proposed a novel statistical model for long read isoform quantification that applies an EM on reads assigned to transcripts while accounting for the base quality and read length. However, the evaluation can be substantiated, and there are several aspects that are not properly described or evaluated at all. While the structure is clear, many sentences have missing citations. The method is available online, however, following the installation instructions and the documentation returned an error.

Major comments:

1. The authors evaluated the performance of LIQA and compared it with FLAIR and Mandalorion. Mandolorion has been published a few years ago, the authors might want to consider more recent methods such as Talon, or the recommended Oxford Nanopore pipeline for comparison (https://github.com/nanoporetech/pipeline-transcriptome-de). There is one concern about the performance of FLAIR, which shows inconsistency between simulated data and real data for UHC runs. It would help if the authors could elaborate on this difference.

2. For the performance evaluation, authors have considered TPM and relative isoform abundance, and used different measures including correlation, and RMSE

1. Given the distribution of TPM and isoform relative abundance are rather skewed, the authors should consider to use Spearman correlation instead of Pearson correlation.

2. It was mentioned that the different measures were computed for different expression levels, gene, isoform and isoform relative, please elaborate on this, as the existing results seem to only show the isoform level.

3. Another point for the results is that the authors have focused on genes with 2 or more isoforms, which constitutes a rather small percentage of total isoforms in usual annotations

1. Please specify the percentage of such number of isoforms in the simulation and real data analysis to assess what is the level of sub-population

2. The authors should consider adding the results for the single isoform genes as supplementary, it might be trivial but it should also be assessed for completeness.

4. The authors have taken into account the fragmented reads by modelling these incomplete reads with a survival model. This is an interesting approach. The definition of full length reads is not clear.

1. The authors consider reads with ending alignment positions (is it both ends or just one ends?) within 20bp of any known isoform as complete. However, this might not be truly a complete read. Please extend the description and re-define this if required.

2. It would be interesting to know what is the distribution of complete and incomplete reads from the simulation and the real data.

5. The authors made a statement in lines 240-241 that the better performance of LIQA is due to factorizing the correction for base quality and read length, and in lines 163 they state that the improvement is due the use of an EM that considers unequal weights for each read to account for mapping bias and uncertainty. While this might be valid, these claims are not supported by data or results. Please analyse the impact of the EM design on quantification by comparing LIQA to a baseline model where base quality and read length are not adjusted and compare it to the existing LIQA results to support this claim.

6. For short read data, the authors could additionally estimate expression using Salmon and Kallisto to demonstrate that the results are robust when another method is used.

7. The results for DAS are largely descriptive, some of the claims are based on examples. Some statements are not shown by results figures or tables (e.g. line 296-297)

8. Many citations are missing for tools, data, and statements.

9. Results presentation could be improved. The sashimi plots with annotation comparison in Figure 5E are difficult to interpret, the annotation could be re-ordered so that it matches between long and short read, labels are very small.

10. The claim that LIQA has an advantage to deal with length bias and 3' bias correction seems to be too strong. The results show an improvement over other methods for longer transcripts, but the results do not specifically evaluate how LIQA deals with length bias or 3' bias.

11. The method is available online, however it could not be run due to an error ("ImportError: No module named bin"). The installation ("git clone") and the documentation are not of a very high standard making it difficult to use for many users. For example, package managers for python (https://pypi.org/) could simplify installation. Automatic tests might catch execution errors. The method seems not to run with a default file format such as GTF or GFF, instead it is suggested to download from the UCSC Browser (even though this is not clear from the documentation, it could be that the file format is identical to GFF in which case this should be documented and files should be names accordingly)

12. How do the authors deal with error correction? FLAIR for example uses error correction before reads are assigned to transcripts

13. How are reads assigned to transcripts? Do the authors require exact matches, or do they allow for some errors or incorrect splice site alignments?

Minor comments:

1. Lines 50-52, citations for alternative splicing events missing

2. Lines 52-53, citation for short read RNA-Seq being used for transcriptomic studies

3. Lines 53-55, "However, due to limited read length (ranges from 50 to 150 nucleotides), transcripts cannot be fully sequenced and characterized by short-read RNA-seq data.". Short reads can fully sequence transcripts, it's just the read assignment might not be accurate.

4. Line 55 "This partial sequencing", I would avoid using partial sequencing as short read RNA-Seq would actually provide coverage for full transcripts

5. Line 57 "gene/isoform" avoid the usage of slash?

6. Lines 59-62 "To tacklet these challenges, a number of computational tools, including RSEM[6], eXpress[7], TIGAR2[8], Salmon[9], Sailfish[10], Kallisto[11], Cufflinks[12], CEM[13], PennSeq[14], IsoEM[15], Stringtie[16], SLIDE[17], iReckon[18] and RD[19].....", It looks like StringTie, SLIDE and iReckon are more focused on novel transcript discovery than quantification? https://bmcgenomics.biomedcentral.com/articles/10.1186/s12864-017-4002-1. There is a type in this sentence.

7. Lines 62-64 "but different bias correction algorithms can result in conflicting estimates. Therefore, fragmented short reads cannot quantify isoform expression accurately, especially at complex gene loci." Add references that support this statement

8. Line 124, citation of NanoSim, should cite this one instead? https://academic.oup.com/gigascience/article/9/6/giaa061/5855462

9. Lines 142-143, shouldn't this be (0,0.75)? IQR should have two values?

10. Lines 145, "demonstrating 3' bias", Is there any plots supporting this?

11. Lines 148-149, "For each simulated dataset, we computed different measurements to evaluate the estimation accuracy of each method", consider change to "for each simulated dataset, we computed a set of measurements to ......". The original sentence sounds like different measurements were computed for different simulated dataset

12. Line 156, "Figure 2(A)", There seems to be a mis-reference in terms of the figure content? Figure 2(A) gives the read length distribution instead? Please check and correct the figures if necessary.

13. Lines 260-261, 63% of mappable reads seems rather low, is there a reference to say that this is high quality?

14. Lines 268, "....at least 50 reads compared…", by setting this threshold, what is the percentage of transcripts remaining? Given 50 reads is a rather high number, the results might be biased for highly expressed isoforms

15. Line 478, "sequence mRNA directly", iis this direct RNA or cDNA sequencing?

**Reviewer 2**

**Are you able to assess all statistics in the manuscript, including the appropriateness of statistical tests used?** Yes

**Comments to author:**

This manuscript presents a new Python-based tool to quantify gene and isoform expression and identify differentially alternative splicing (DAS) events based on long-read RNA-seq data.

The authors claim that LIQA improves the accuracy of isoform expression quantification from long-read RNA-seq data, in comparison with programs developed to deal with short-read (e.g., RSEM, Salmon, and Kallisto) and other tools specifically designed for the analysis of long-read RNA-seq data (such as FLAIR and Mandalorion). That improvement is achieved through the assignment of different weights across reads mapping to each isoform, based on their base-pair quality scores and on isoform-specific read length information obtained after aligning RNA-seq data using popular tools for the purpose (e.g. minimap2 and STAR). Those weights also account for truncated reads and potential coverage bias towards the 3´ end of transcripts from specific Nanopore protocols. In summary, LIQA allows users to get improved gene and isoform abundance estimates from long-read RNA-seq data.

The authors benchmarked the results of running LIQA on simulated and real datasets against those of established tools (FLAIR and Mandalorion) and showed that their tool particularly outperforms others when there is the need to correct for the aforementioned 3´ end bias.

We praise the authors for a well-structured manuscript, the clever usage of established mathematical models (such as analysing read length as censored data to apply Kaplan-Meier estimators in the quantification of truncated or incomplete reads), and developing an interesting tool that builds upon state-of-the-art approaches. The organisation and separation between methodological aspects in Methods and Materials is very intelligible, with the main text also enabling a non-expert reader to grasp the critical features of LIQA without having to initially go through the all methodological intricacies.

However, as detailed below, there are several shortcomings in the rigour with which results are presented and interpreted. These need to be addressed before we can consider this work sound enough for publication.

MAJOR CONCERNS:

1. Several of the plots supporting the results lack enough details for objective examination. For example, the bar plots summarising Spearman's coefficients and RMSE values (e.g., Figures 2 and 3, Supplementary Figures 3 to 6) should include the actual values (for instance, on top of the respective bar), allowing the reader to unbiasedly assess the magnitude of differences between tools.

2. More importantly and related to the previous point, the results are generally described with a bias towards LIQA that is typically only supported by subjective examination of plots. For instance, the usage of "clearly" is too strong when results only slightly favour LIQA. We urge the authors to avoid subjective overstatements and empirically and/or statistically support their observations. For instance, when describing the results in figure 3(B), in lines 180-181, the authors claim that "LIQA appears to be much more accurate than the other two methods" while visual inspection suggests that the difference in Spearman's correlation between LIQA and FLAIR is roughly equal or smaller than that found for panel A, where FLAIR is actually more accurate than LIQA for shorter isoforms. Moreover, the significance of the reported differences in correlation should be statistically tested and the respective p-values should be included in the respective figures.

3. In lines 240-242, it is claimed that the alleged better performance of LIQA is due to accounting for isoform length bias and base quality scores. Although plausible, this is never demonstrated. For instance, the differences in correlation of TPM between tools do not exhibit an obvious isoform length-associated trend. The actual performance of the benchmarked tools needs to be profiled against different levels of length bias and qualitatively related with base quality scores for the merits of accounting for those effects to be stated.

4. The results illustrated in Figure 5(E) are misinterpreted (lines 307-312). From the sashimi plots therein, one cannot conclude that the exon in the green square is higher in cancerous cells than in normal-like ESCC. Careful examination suggests a much lower coverage for the normal samples, particularly in the 5´ half of the transcript, clearly not enough to resolve that exon (read coverage in the vicinity is of only 3 reads, compared with >10 for the cancerous sample and dozens for the short-read data). The same lack of enough coverage to resolve DAS for the exon in the red box applies. In this example, there's no evidence for the alleged "flip-flop expression pattern" and, contrary to claimed by the authors (lines 310-312), the long-read data performs worse than short-read data, for which read coverage is much higher.

5. In the Discussion, the authors describe their concerns about finding the right tradeoff between robustness and running time, suggesting that future versions of LIQA may sacrifice the former to favour the latter. This reflection should be supported by precise information on the running time differences between the tools being compared. The running time of LIQA should be disclosed. How slower is LIQA than the other tools? Minutes? Hours? This is an important piece of benchmarking information. The decision of relaxing the robustness to get a faster analysis should ideally be tuned by the user, if sufficient information on this tradeoff is provided.

6. The whole DAS section is oversimplified, as only numbers of DAS events are compared, even though the precise profiling of such events is where the usage of long-read technology can prove advantageous. The precision of LIQA in quantifying the relative isoform abundance associated with DAS events (and not only the ability to detect them) should also be evaluated.

MINOR ESSENTIAL REVISIONS:

7. LIQA should be more easily installable as a Python package via pip (instructions on how to do so in Python's official documentation available at https://packaging.python.org/tutorials/packaging-projects).

8. Supplementary figures (except for Supplementary Figure 1) are not mentioned in the manuscript. We agree with the inclusion of those supplementary figures but they should be properly referenced across the manuscript.

9. In line 225-228, the authors mention that, when applying LIQA to universal human reference RNA-seq data, they decided to filter out from the qRT-PCR measurements genes with a single isoform "because estimating isoform-specific expression for these single-transcript genes is trivial". Although this may be true, a typical user will also be interested in these unique isoform genes and they would still serve as positive controls for LIQA's performance. They should therefore be included the comparison, marked as a separate group.

10. In the Abstract, lines 31-32, sentence "Moreover, given isoform usage estimates, (...)" does not convey that LIQA is able to provide these values and may lead to confusion on whether the user needs to provide LIQA with such data.

11. In the Abstract, lines 32-34, in sentence "We evaluated (...) and demonstrated that it [LIQA] outperforms other approaches in rare isoform characterization (...)", it is not clear 1) what "rare" means in this context and 2) how this was quantified, as no support for such observation can be found in the manuscript.

12. In the Introduction, lines 53-55, in sentence "However, due to limited read length (ranges from 50 to 150 nucleotides) transcripts cannot be (...)" the presented length range definition of short reads is over-simplistic, given the intention of motivating the usage of long reads. Short reads can be 200nt-long and still lack the properties of longer reads. Moreover, providing an estimate for the range of mRNA transcripts can help illustrating the convenience of using long reads. This would also facilitate the interpretation of "allows a large portion or the entire mRNA or cDNA molecule to be sequenced" in line 72.

13. In the Introduction, lines 63-64, the fact that "different bias correction algorithms can result in conflicting estimates" does not mean that "(...) fragmented short reads cannot quantify isoform expression accurately (...)". Although results can be conflicting, some programs may accurately estimate isoform expression. Such bold statement would need support from rigorous benchmarking.

14. In the Introduction, lines 73-76, supporting references for the contribution of long-read RNA-seq to "rare isoform discovery, isoform expression quantification and DAS event detection" are missing and the mention to the possibility of detecting RNA modifications based on the "success of DNA modification detection" (also missing a reference) is speculative and does not positively contribute to the manuscript's flow.

15. In the Introduction, lines 77-79, sentence "However, there are still a few unique challenges to analyze long-read RNA-seq data because existing methods developed for Illumina short-read RNA-seq do not have optimal performance when directly used on long-read RNA-seq." is lacking a reference. In the absence of a reference, an appropriate justification for the assumptions underlying the non-applicability of short-read RNA-seq methods to long reads is required.

16. In the Introduction, lines 100-101, sentence "Our results demonstrate that LIQA outperforms existing methods in isoform expression quantification." is a misleading overstatement, given that is not descriptive enough. LIQA does not outperform existing methods in all conditions.

17. Although the manuscript's Figure 1 version is more appealing aesthetically, the directionality provided in GitHub's version of the same flowchart illustrating LIQA is more informative on how the program works. In the manuscript's Figure, the top and bottom boxes in panel A suggest two different levels of information/data types. Moreover, it is not clear what the input and output of each step of analysis are. For instance, the flowchart misleadingly suggests that LIQA aligns raw RNA-seq data.

18. Results, line 107: it is not clear what "read length probability" refers to at this stage of the manuscript.

19. Figure 2 and Supplementary Figure 1 would be more comparable if the X-axes are kept the same across the plots. The numbers indicating the median read lengths and median count in each of the downsampling libraries should also be included in the plots in Supplementary Figure 1 (like in Figure 2A).

20. In Results, lines 143-145, the mention to "the coverage plots of the simulated data" should be accompanied by a reference to those plots, which are to be shown as Supplementary Figures.

21- It is not clear what distributions are represented in the boxplots for the DAS statistics (recall, precision and F1 score) in Figure 3C. Do these refer to 10 different simulations? If so, points corresponding to the 10 values should be part of the plot as well. Also, the line width used is in those plots is too thick for the reader to discriminate quartiles.

22. Figures 4(A) and 4(B) and their captions could be improved. What the red line in panel A is should be explicitly stated. The colours of dots in panel A should match those of the legend and of panel B. The colour legend should be closer to panel B. The Y-axis label is missing in panel B.

23. In Figure 5(E), "Long-read" and "Short-read" should be the actual titles of the two panels and not be misleadingly placed near the representation of the annotated isoforms.

24. Regarding the Kaplan-Meier plots in Supplementary Figures 7 to 9, the authors should consider marking the isoform length in the plots to facilitate interpretation.

25. Supplementary Figures 7 and 8 should have at least some genes in common, for comparison.

Although the first paragraph of Methods is written with clarity and we congratulate the authors for introducing the mathematics with intelligible explanations, the meaning of ZR,I (line 380) is not explained.

26. Methods, lines 407-408, "The read is treated as complete when the distance between its ending alignment position and any known isoform 5' end is less than 20 bp (Figure 1(B)).": the authors should explain the choice of a threshold of 20 bp and include this information in the figure scheme.

PROOFREADING EDITS:

27. Line 59: "tackle" instead of "tackle".

28. Line 107: "corrects" instead of "correct".

29. Line 114: "expression" instead of "expressions".

30. Line 156: Figure 2(A) should be 2(B,C) and, similarly, Figure 2(B) in line 160 should be 2(C).

31. Line 162: "higher" should be "lower", as this refers to the RMSE.

32. Line 191: "we sampled relative abundances of isoforms" instead of "we sampled isoform relative abundances of isoforms".

33. Line 201: "outperform" instead of "outperforms".

34. Line 211: "hardly a full" instead of "hard to be full".

35. Line 224: "gold" instead of "golden".

36. Line 238: one "only" should be removed at the end of the sentence.

37. Line 240: "major reason for the better performance of LIQA" instead of "major reason of the better performance for LIQA".

38. Lines 245-248: "severe 5´ degradation" instead of "severe 3' degradation" and "Terminal exons at the 5´ end in the red square" instead of "Terminal exons at 3' end in red square.

39. Line 255: "Acute myeloid leukemia (AML)" instead of "AML", as the acronym needs to be introduced.

40. Line 257: URL https://denbi-nanopore-training-course.readthedocs.io/en/latest/basecalling/basecalling.html%23references returns error "This page does not exist yet." and should be fixed.

41. Line 264: "analyze short-read sequencing data for the same AML sample" instead of "analyze a short-read data sequenced based on the same AML sample".

42. Line 265: "reads" instead of "read".

43. Line 271: "surprising" instead of "surprised".

44. Line 288: "differences" instead of "difference".

45. Line 296: "most frequent type of event" instead of "richest event".

46. Line 298: "type" instead of "types".

47. Line 325: "LIQA is more effective" instead of "LIQA is effective".

48. Line 339: "files" instead of "file".

49. Line 346: "read coverage" instead of "reads covered".

50. Line 348: "may be" instead of "maybe".

51. Line 354: "per-base" instead of "per-based".

52. Line 411: "isoforms are treated" instead of "isoforms is treated".

53. Line 425: "defined" instead of "deifned".

54. Legends of Supplementary Figures 10 and 11: it must be stated what red and blue Sashimi plots correspond to.

**Reviewer 3**

**Are you able to assess all statistics in the manuscript, including the appropriateness of statistical tests used?** No

**Comments to author:**

The authors present a novel statistical method, LIQA, for isoform-level quantification and differential splicing detection from long read RNA-Seq data, such as that generated by Oxford Nanopore (ONT) and Pacific Biosciences (PacBio). The novelty of LIQA's quantification approach relative to competing methods is that it attempts to model the uncertainty of the assignment of each long read to its isoform of origin. Critical aspects of the statistical model include a model of read lengths that takes into account the phenomenon of long reads that are truncations of full transcripts and a model of the sequence similarity (referred to as the "quality score") between a long read and a candidate isoform. LIQA performs quantification by finding maximum likelihood estimates of its statistical model, via the EM algorithm.

LIQA's performance is compared to two competing methods, FLAIR and Mandalorion, using both simulated data and real data from the well characterized Universal Human Reference (UHR) RNA sample. Results indicate that LIQA generally performs better than the other two methods. In addition, two case studies of using LIQA on data from AML and ESCC samples are presented.

In general, a more rigorous statistical treatment of isoform quantification from long read data is a welcome contribution. And the breadth of data sets on the methodology is evaluated is excellent. My major concerns with the current manuscript are with regard to the evaluation criteria used and lack of justifications for the components of the model used by LIQA.

Major comments:

1. From what I can understand from the manuscript, each long read is mapped to one gene, but there may be ambiguity as to which isoform of the gene the long read is derived from. Therefore, the only aspects of the quantification to be evaluated are the relative abundances of the isoforms within each gene. The evaluations of "global" gene expression or isoform expression are not informative, or are at least redundant with the evaluation of the within-gene relative abundances. I would recommend restricting evaluations to the within-gene relative abundances. In addition, it does not seem proper to pool relative abundances from isoforms across all genes and compute correlations; the unit of evaluation is really at the gene level, since the within-gene relative abundances of the isoforms are highly correlated. I would recommend computing an accuracy metric for each gene that represents how well the isoforms of that gene are ranked/quantified, and then summarizing these gene accuracies.

2. A number of aspects of the quantification model are unclear:

(A) why is the probability of a read originating from an isoform proportional to the product of the isoform length and the isoform abundance? It is clear why the length factors into this for short-read RNA-seq data due to the fragmentation aspects of the protocol, but it is not clear why this is a factor in long-read (often full-length) RNA-seq data.

(B) It does not appear that the read quality scores take into account indels. Is that the case? If so, this seems odd given how common indels are in long-read data, especially ONT.

(C) The generative model for the read length is a bit odd, in that it is defined via Bayes rule and it seems to be constructed by assuming that we draw from a read length distribution first, and then select among the isoforms that are larger than that read length. That seems counter to the generative process of the sequencer, which would first select the transcript and then generates the read given that transcript.

(D) Are reads only allowed to be truncated at the 5' end, or are 3' truncations allowed as well?

3. In the evaluation of DAS, it is unclear how the predictions for the various methods were thresholded, i.e., was a FDR of 0.05 targeted? It would be more informative to show a precision-recall curve here with the point obtained with the given threshold highlighted.

4. It is unclear how the DAS methodology differs from that of the other methods. More discussion of this is warranted. In addition, could the DAS methodology presented here be applied to the outputs of the other quantification methods, or is it specific to the output of LIQA?

Minor comments:

4. Figure 1A - I find this diagram to be confusing. It would be clearer if there could be two types of objects in this diagram, processes (algorithms) and inputs/outputs (data), with the inputs and outputs of each process clearly shown and linked together.

5. "Second, we compared the accuracy statistics for 5' terminal exon and 3' terminal exon of each isoform (Figure 3(B))" What is being measured at these exons?

6. "estimation accuracy of all methods is lower than simulated data because the qRT-PCR measures may not be accurate,": I'm not sure it is fair to directly fault the qRT-PCR measurements here; estimation accuracy could easily be worse simply because this is real data.

**Authors Response**

**Point-by-point responses to the reviewers’ comments:**

***Reviewer #1: The authors present an isoform expression quantification method that performs weighted quantification using long reads with an EM algorithm. They demonstrate good performance in isoform quantification and differential splicing analysis with simulated datasets and they further evaluated quantification with real long read data.***

***This paper touches on an important and interesting topic to propose a novel quantification method for isoform expression with long read RNA-Seq. The authors proposed a novel statistical model for long read isoform quantification that applies an EM on reads assigned to transcripts while accounting for the base quality and read length. However, the evaluation can be substantiated, and there are several aspects that are not properly described or evaluated at all. While the structure is clear, many sentences have missing citations. The method is available online, however, following the installation instructions and the documentation returned an error.
 
Major comments:***

***1. The authors evaluated the performance of LIQA and compared it with FLAIR and Mandalorion. Mandolorion has been published a few years ago, the authors might want to consider more recent methods such as Talon, or the recommended Oxford Nanopore pipeline for comparison (***[***https://github.com/nanoporetech/pipeline-transcriptome-de***](https://github.com/nanoporetech/pipeline-transcriptome-de)***). There is one concern about the performance of FLAIR, which shows inconsistency between simulated data and real data for UHC runs. It would help if the authors could elaborate on this difference.***

Thanks very much for your constructive suggestions. We conducted additional analysis of simulation data and the UHR data, using both the TALON and Oxford Nanopore pipeline, as suggested. LIQA, FLAIR and TALON have higher Spearman’s correlation and lower RMSE values than Mandalorion and the Oxford Nanopore pipeline across all datasets. As shown in Figure 2 and 3, on average, LIQA outperformed TALON and Oxford Nanopore pipeline with 5.5% and 13.2% higher Spearman’s correlation, and 7.6% and 12.2% lower RMSE. LIQA performed especially well compared to other methods for genes with lower sequencing depth/coverage. The less accurate estimates of TALON and Oxford Nanopore pipeline might be due to the lack of consideration of the read quality scores. TALON uses discrete read count of transcript for isoform expression estimation and the Oxford Nanopore pipeline indeed uses Salmon, a short-read approach, to quantify isoform usage. For FLAIR, the reason of less accurate performance in UHR dataset is due to limited read count. Instead of estimating a continuous value, FLAIR provides discrete estimations by directly counting the number of reads aligned to each corresponding gene or isoform. We examined the intermediate file “counts_matrix_rpg.tsv” of FLAIR and found that 65% (85/132) isoforms have read coverage less than 3, yielding less accurate estimates for UHR data. Also, as shown in Figure 2, FLAIR has 10% lower Spearman’s correlation than LIQA when read coverage is low (0.5). This difference is much more significant compared to simulation data with higher sequencing depth (difference = 2%, read coverage = 5M reads). We have revised the manuscript and updated description in the Results section (page 7-10), as well as Figure 2, 3, 4. Hopefully, by incorporating the new results on two additional computational approaches, the benchmark study is now clearer and more comprehensive. We thank the reviewer for suggesting these additional benchmarking studies.

***2. For the performance evaluation, authors have considered TPM and relative isoform abundance, and used different measures including correlation, and RMSE***

1. ***Given the distribution of TPM and isoform relative abundance are rather skewed, the authors should consider to use Spearman correlation instead of Pearson correlation.***

Thank you for pointing this out. We have updated the summary statistics in Figures 2-5 to Spearman’s correlation to measure the performance accounting for the skewed distribution of isoform usage estimates.

***2. It was mentioned that the different measures were computed for different expression levels, gene, isoform and isoform relative, please elaborate on this, as the existing results seem to only show the isoform level.***

We apologize for the lack of clarity on expression levels. “Isoform-specific gene expression”, which was mentioned in the manuscript, is equivalent to isoform expression. Essentially, LIQA is developed to assign a long read to its isoform of origin because there is ambiguity as to which isoform the long read is derived from. Therefore, to benchmark the performance of LIQA, we evaluated the accuracy of isoform expression estimates in the Results section, including relative expression level (relative abundance) and absolute expression level (TPM, RPG). We have revised the manuscript and added explanations on “isoform-specific gene expression” in the Introduction section, and hopefully this term is now clearer to the reviewer and readers.

For your suggestion regarding comparison at the gene level, we considered two ways to benchmark the performance. First, we have summarized the Spearman’s correlation between estimated and true TPM at gene-level in the Supplementary Figure 4(B)(left). Second, we used Hellinger distance to quantify the distance between underlying isoform relative abundance and estimates for each gene. Hellinger distance is a measure that describes the similarity between two probability distributions. Specifically, for gene with isoform relative abundance $\boldsymbol{\theta}=(\theta_{1},\ldots,\theta_{I})$ and $\hat{\boldsymbol{\theta}}=(\hat{\theta}_{1},\ldots,\hat{\theta}_{I})$, their Hellinger distance is defined as $H\left( \boldsymbol{\theta},\hat{\boldsymbol{\theta}} \right)=\frac{1}{\sqrt{2}}\sqrt{\sum_{i=1}^{I} \left( \sqrt{\theta_{i}}-\sqrt{\hat{\theta}_{i}} \right)^{2}}$ (Supplementary Figure 4(B)(right)). Our results indicate that LIQA still yields more accurate expression estimates at the gene level.

***3. Another point for the results is that the authors have focused on genes with 2 or more isoforms, which constitutes a rather small percentage of total isoforms in usual annotations***

***1. Please specify the percentage of such number of isoforms in the simulation and real data analysis to assess what is the level of sub-population***

Thanks for pointing this out. In total, there are 84,818 isoforms based on GENCODE annotation, among which 27,793 (32.7%) are from genes with only one isoform and 57,025 (67.2%) are from genes with 2 or more isoforms. We have revised the manuscript and added this description to illustrate the level of sub-population in the Results section.

***2. The authors should consider adding the results for the single isoform genes as supplementary, it might be trivial but it should also be assessed for completeness.***

Thanks for the suggestions. We conducted additional simulations and real data analyses for genes with only one isoform. As all isoform relative abundances equal to 1, we measure the similarity between estimated TPM and the ground truth using Spearman’s correlation. As shown in Supplementary Figure 4(A), all methods have significantly improved performance for isoform usage quantification. This is not surprising because expression quantification of genes with a single isoform is much easier than genes with 2 and more isoforms. All methods compute TPM based on gene-level read count without conducting read deconvolution process.

***4. The authors have taken into account the fragmented reads by modelling these incomplete reads with a survival model. This is an interesting approach. The definition of full length reads is not clear. The authors consider reads with ending alignment positions (is it both ends or just one ends?) within 20bp of any known isoform as complete. However, this might not be truly a complete read. Please extend the description and re-define this if required.***

Thanks for pointing this out. LIQA utilizes statistical survival model to account for 3’ sequence bias. Therefore, we only consider reads with ending alignment position at the 5’ end. Due to alignment and sequencing errors, ending positions of reads might not be consistent with annotated isoform boundary. To classify complete and incomplete reads accounting for misalignment, we treat a read as complete when the distance between its ending alignment position and any known isoform 5’ end is less than a tolerance threshold (default = 20 bp) in our analysis. We tested the sensitivity of LIQA to this threshold when estimating read length probability using simulated data. 20 bp is a robust distance for read-isoform assignment. Compared to exact-matched complete read (tolerance error = 0 bp), the effect on read length probability curve estimation is trivial. To make our method more flexible, we modified LIQA program to allow user to specify this threshold in analysis (default tolerance threshold = 20 bp). We have revised the manuscript accordingly (Figure 1(A)) and updated the program on GitHub as well.

***5. It would be interesting to know what is the distribution of complete and incomplete reads from the simulation and the real data.***

Thanks for pointing this out. Following your suggestion, we have summarized the distribution of complete and incomplete reads in Supplementary Figure 8(C). For three real datasets, ~70% of reads completely covered the annotated isoforms. Most of incomplete reads (~20%) covered at least 50% of nucleotides for the isoform while others covered less than 50%.

***6. The authors made a statement in lines 240-241 that the better performance of LIQA is due to factorizing the correction for base quality and read length, and in lines 163 they state that the improvement is due the use of an EM that considers unequal weights for each read to account for mapping bias and uncertainty. While this might be valid, these claims are not supported by data or results. Please analyse the impact of the EM design on quantification by comparing LIQA to a baseline model where base quality and read length are not adjusted and compare it to the existing LIQA results to support this claim.***

Thanks very much for your constructive suggestions. We agree that it is important to assess the robustness of EM-design LIQA to low read quality and read length bias. The motivation of using EM algorithm is to have accurate estimation of isoform usage accounting for ambiguity of reads’ isoform origin. Following your suggestion, we have conducted a new study based on the simulation data using four different models of LIQA as following:

Full model that accounts for both read length bias and read quality score:

$$L\left( \tilde{\boldsymbol{\Theta}} | \mathbf{R},\mathbf{Z} \right)=\prod_{r\in\boldsymbol{R}} \prod_{i\in\boldsymbol{I}} \left( q\left( r,i \right)\cdot f\left( L_{r} \right| iso. =i)\tilde{\theta}_{i} \right)^{\boldsymbol{Z}_{\boldsymbol{R},\boldsymbol{I}}\left( r,i \right)}$$

Read length model that accounts for read length bias but not read quality score:

$$L\left( \tilde{\boldsymbol{\Theta}} | \mathbf{R},\mathbf{Z} \right)=\prod_{r\in\boldsymbol{R}} \prod_{i\in\boldsymbol{I}} \left( f\left( L_{r} \right| iso. =i)\tilde{\theta}_{i} \right)^{\boldsymbol{Z}_{\boldsymbol{R},\boldsymbol{I}}\left( r,i \right)}$$

Read quality model that accounts for read quality score but not read length bias:

$$L\left( \tilde{\boldsymbol{\Theta}} | \mathbf{R},\mathbf{Z} \right)=\prod_{r\in\boldsymbol{R}} \prod_{i\in\boldsymbol{I}} \left( q\left( r,i \right)\cdot\tilde{\theta}_{i} \right)^{\boldsymbol{Z}_{\boldsymbol{R},\boldsymbol{I}}\left( r,i \right)}$$

Baseline model without bias correction:

$$L\left( \tilde{\boldsymbol{\Theta}} | \mathbf{R},\mathbf{Z} \right)=\prod_{r\in\boldsymbol{R}} \prod_{i\in\boldsymbol{I}} \left( \tilde{\theta}_{i} \right)^{\boldsymbol{Z}_{\boldsymbol{R},\boldsymbol{I}}\left( r,i \right)}$$

Figure 3(C)(D) show the comparison of isoform estimation accuracy using different models. For isoforms with more non-uniform read coverage (Chi-square statistics > median), model with read length bias correction has 9% (Full model vs read quality model) and 10% (Read length model vs baseline model) higher Spearman’s correlation. For genes with less average read quality, model with read quality score has 6% higher Spearman’s correlation. Isoform estimation accuracy drops noticeably when using model with no read-length bias correction. These results demonstrate the benefit of modeling read quality and coverage bias. We have revised the manuscript and added these evaluations in Results section (page 9 paragraph 2).

***7. For short read data, the authors could additionally estimate expression using Salmon and Kallisto to demonstrate that the results are robust when another method is used.***

Thanks for the suggestion. We conducted additional analysis of UHR data using Salmon and Kallisto (Supplementary Figure 8(B)). For short-read approaches, Cufflinks seems to correlate with the qRT-PCR measurements better than CEM, Salmon and Kallisto. Comparison of different methods using RMSE reveals a similar pattern.

***8. The results for DAS are largely descriptive, some of the claims are based on examples. Some statements are not shown by results figures or tables (e.g. line 296-297)***

We apologize for the lack of details on DAS analysis results. Following your suggestion, we included more summary statistics and figures in the Results section of the revised manuscript. To compare the performance of LIQA with other methods on DAS detection, we first generated ROC curve and precision-recall curve. As shown in Figure 3(E), LIQA achieved AUC = 0.94 after FDR control (FDR < 0.05). Given an FDR threshold of 0.05, LIQA gave the best performance with precision = 0.98 and recall = 0.78. Compared to LIQA, the second-best performer FLAIR yields 0.1%, 0.3%, 3.5% less in precision, recall and AUC, respectively. Second, we examined isoform relative abundance accuracy from correctly and incorrectly detected DAS genes by LIQA. After FDR control, we identified 537 out of 2,465 genes to show DAS, of which 431 are true positives and 1836 are true negatives. For these correctly predicted genes, true isoform relative abundance is highly correlated with LIQA’s estimates (Spearman’s correlation= 0.91). For false positive and negative genes, the Spearman’s correlation is 38% lower than true positive and negative genes. This is not surprising because accurate estimation of isoform expression level leverages the power of regression model in detecting DAS events. We have revised the manuscript and added these analysis results to Results section (page 9-10).

***9. Many citations are missing for tools, data, and statements.***

We apologize for missing citations. Following your comments, we have added numbers of citations for tools, data and statements. Specifically, we have added Pan et al.[1] and Wang et al.[2]’s work as references for alternative splicing events. We have added Wang et al.[3]’s work as reference for short read RNA-Seq being used for transcriptomic studies. We have added Hu et al[4], Steijger et al[5] and Tilgner et al[6]’s work as references to support the statement of bias correction difference for short-read approaches. We have changed the reference of NanoSim[7]. We have cited Au et al.[8], Clark et al.[9], Sharon et al.[10], Gupta et al.[11] and Tilgner et al.,[12]’s work as reference to support the statement of isoform characterization using long-read data.

***10. Results presentation could be improved. The sashimi plots with annotation comparison in Figure 5E are difficult to interpret, the annotation could be re-ordered so that it matches between long and short read, labels are very small.***

Following your suggestion, we have re-ordered the annotation and the figure panels now match between long-read and short read data. Also, as suggested, we increased the size of label to facilitate interpretation.

***11. The claim that LIQA has an advantage to deal with length bias and 3' bias correction seems to be too strong. The results show an improvement over other methods for longer transcripts, but the results do not specifically evaluate how LIQA deals with length bias or 3' bias.***

We apologize for the lack of evaluation on how LIQA deals with length bias or 3’ bias. Following your suggestion, we evaluated the impact of degree of non-uniformity in read coverage on isoform estimation. We considered the chi-square statistics that measures the goodness-of-fit of coverage uniformity. Isoforms were divided into two categories based on median of the corresponding measure (Chi-square statistic > median, Chi-square statistic < median). Isoform expression with non-uniform read coverage is more challenging to estimate because the 5’ end is less likely to be covered by sequencing reads compared to 3’ end. Figure 3(B) shows the comparison of Spearman’s correlation for five methods. For isoforms with length less non-uniform read coverage, Spearman’s correlations of LIQA and FLAIR are close. However, despite of reduced Spearman’s correlation value, LIQA improved estimation accuracy compared to other four methods for isoforms with more non-uniform read coverage (Chi-square statistic > median). The improvement of LIQA compared to FLAIR is 5% higher for these isoforms. We observed similar pattern when accuracy was quantified by RMSE. This is because LIQA models potential truncated reads that result in 3’ coverage bias when quantifying isoform expression. We have revised the manuscript and added this description in the Results section.

***12. The method is available online, however it could not be run due to an error ("ImportError: No module named bin"). The installation ("git clone") and the documentation are not of a very high standard making it difficult to use for many users. For example, package managers for python (***[***https://pypi.org/***](https://pypi.org/)***) could simplify installation. Automatic tests might catch execution errors. The method seems not to run with a default file format such as GTF or GFF, instead it is suggested to download from the UCSC Browser (even though this is not clear from the documentation, it could be that the file format is identical to GFF in which case this should be documented and files should be names accordingly)***

Thanks for pointing this out. Following your suggestion, we have substantially improved the program and documentation for LIQA. Specifically, we have published LIQA to PyPi to simplify installation for users. Also, we have changed the isoform annotation input file format to GTF. Moreover, we are making Conda as well as docker image for LIQA. A few users already used LIQA and have communicated feedback to us or submitted GitHub issues to us. The usability of LIQA has been significantly improved now.

***13. How do the authors deal with error correction? FLAIR for example uses error correction before reads are assigned to transcripts***

Thanks for pointing this out. LIQA does not use error correction of long reads (it is typically used in genome assembly studies when high coverage data is available). If short-read data is available, there are 3rd party tools to do error correction on long-read data. However, if the “error correction” term from the reviewer refers to “boundary inference” for intron/exon boundaries, then it can be addressed by post processing the alignment data using LIQA. LIQA allows some errors in alignment for read-isoform assignment. Given a reference annotation file, LIQA allows user to specify the maximum number of incorrect alignment base pairs. In addition, we quantify isoform expression accounting for read-based quality score difference. LIQA assigns more weight to reads with higher quality score in the likelihood function, then yields more accurate estimates using EM-algorithm.

***14. How are reads assigned to transcripts? Do the authors require exact matches, or do they allow for some errors or incorrect splice site alignments?***

We apologize for the lack of clarity on read-isoform assignment. LIQA allows some errors in alignment when calculating read-isoform compatibility matrix $\boldsymbol{Z}_{\boldsymbol{R},\boldsymbol{I}}$. Given a reference annotation file, LIQA allows user to specify the maximum number of incorrect alignment base pairs. For read $r$ and isoform $i$, $\boldsymbol{Z}_{\boldsymbol{R},\boldsymbol{I}}\left( r,i \right)=1$ if the number of mismatches to isoform $i$ for long-read $r$ is less than the incorrect alignment threshold, and $\boldsymbol{Z}_{\boldsymbol{R},\boldsymbol{I}}\left( r,i \right)=0$ if the number of mismatches to isoform $i$ is greater and equal to the incorrect alignment threshold. We have revised the manuscript and added these descriptions in the Methods section (page 18 paragraph 1). Hopefully, this method is now clearer to the reviewer and readers.

***Minor comments:***

***1. Lines 50-52, citations for alternative splicing events missing***

Thanks for pointing this out. We have added Pan et al[1] and Wang et al[2]’s work as references for alternative splicing events.

***2. Lines 52-53, citation for short read RNA-Seq being used for transcriptomic studies***

Thanks for pointing this out. We have added Wang et al[3]’s work as reference for short read RNA-Seq being used for transcriptomic studies.

***3. Lines 53-55, "However, due to limited read length (ranges from 50 to 150 nucleotides), transcripts cannot be fully sequenced and characterized by short-read RNA-seq data.". Short reads can fully sequence transcripts, it's just the read assignment might not be accurate.***

Thank you. We have corrected this sentence to “However, due to limited read length (ranges from 50 to 150 nucleotides), transcripts cannot be accurately characterized by short-read RNA-seq data.”.

***4. Line 55 "This partial sequencing", I would avoid using partial sequencing as short read RNA-Seq would actually provide coverage for full transcripts***

Thank you. We have replaced “partial” with “fragmented” in this sentence.

***5. Line 57 "gene/isoform" avoid the usage of slash?***

Thank you. We have removed all slash signs in the manuscript and corrected them to “or”.

***6. Lines 59-62 "To tacklet these challenges, a number of computational  tools, including RSEM[6], eXpress[7], TIGAR2[8], Salmon[9], Sailfish[10], Kallisto[11], Cufflinks[12], CEM[13], PennSeq[14], IsoEM[15], Stringtie[16], SLIDE[17], iReckon[18] and RD[19].....", It looks like StringTie, SLIDE and iReckon are more focused on novel transcript discovery than quantification?*** [***https://bmcgenomics.biomedcentral.com/articles/10.1186/s12864-017-4002-1***](https://bmcgenomics.biomedcentral.com/articles/10.1186/s12864-017-4002-1)***. There is a type in this sentence.***

Thanks for pointing this out. We agree that StringTie, SLIDE and iReckon are more focused on novel transcript discovery than isoform quantification accounting for sequencing bias. Therefore, we removed these three methods from this sentence which is related with bias correction in isoform quantification.

***7. Lines 62-64 "but different bias correction algorithms can result in conflicting estimates. Therefore, fragmented short reads cannot quantify isoform expression accurately, especially at complex gene loci." Add references that support this statement***

Thank you. We have added Hu et al[4], Steijger et al[5] and Tilgner et al[6]’s work as references to support this statement.

***8. Line 124, citation of NanoSim, should  cite this one instead?***

[***https://academic.oup.com/gigascience/article/9/6/giaa061/5855462***](https://academic.oup.com/gigascience/article/9/6/giaa061/5855462)

Thanks for pointing this out. We have changed the reference of NanoSim to this paper[7].

***9. Lines 142-143, shouldn't this be (0,0.75)? IQR should have two values?***

Thanks. Yes, we have changed IQR to (0.002, 0.75).

***10. Lines 145, "demonstrating 3' bias", Is there any plots supporting this?***

Thank you. We have added the sashimi plot of simulated data to Supplementary Figure 1(B) which demonstrates 3’ bias of long-read RNA-seq data.

***11. Lines 148-149, "For each simulated dataset, we computed different measurements to evaluate the estimation accuracy of each method",  consider change to "for each simulated dataset, we computed a set of measurements to ......". The original sentence sounds like different measurements were computed for different simulated dataset***

Thanks for pointing this out. We have corrected this sentence to “For each simulated dataset, we computed a set of measurements to evaluate the estimation accuracy of each method.”.

***12. Line 156, "Figure 2(A)", There seems to be a mis-reference in terms of the figure content? Figure 2(A) gives the read length distribution instead? Please check and correct the figures if necessary.***

Thank you for pointing out this typo. We have corrected this figure reference.

***13. Lines 260-261, 63% of mappable reads seems rather low, is there a reference to say that this is high quality?***

We have changed this sentence to “We aligned the data against a reference genome (hg38) using minimap2, and 63% long-reads (73% bases) are mapped.”

***14. Lines 268, "....at least 50 reads compared…", by setting this threshold, what is the percentage of transcripts remaining? Given 50 reads is a rather high number, the results might be biased for highly expressed isoforms***

Thanks for pointing this out. The percentage of genes with 50 reads is 51%. It is not surprising that the Spearman’s correlation between estimates using long-read and short-read data is significantly improved for genes with at least 50 reads. To remove this selection bias, we have examined the estimations for all genes as a control group. As shown in the left panel of Figure 5(A), the scatter plot between long-read and short-read estimates for all genes also indicates a good consistency with Spearman’s correlation equals to 0.65.

***15. Line 478, "sequence mRNA directly", iis this direct RNA or cDNA sequencing?***

Yes, we indeed sequenced the Universal Human Reference sample using Nanopore direct mRNA sequencing, which is unique to the Nanopore sequencing technology. We sequenced the other cancer samples by cDNA sequencing, which is more commonly used in RNA-Seq experiments.

***Reviewer #2: This manuscript presents a new Python-based tool to quantify gene and isoform expression and identify differentially alternative splicing (DAS) events based on long-read RNA-seq data.***

***The authors claim that LIQA improves the accuracy of isoform expression quantification from long-read RNA-seq data, in comparison with programs developed to deal with short-read (e.g., RSEM, Salmon, and Kallisto) and other tools specifically designed for the analysis of long-read RNA-seq data (such as FLAIR and Mandalorion). That improvement is achieved through the assignment of different weights across reads mapping to each isoform, based on their base-pair quality scores and on isoform-specific read length information obtained after aligning RNA-seq data using popular tools for the purpose (e.g. minimap2 and STAR). Those weights also account for truncated reads and potential coverage bias towards the 3´ end of transcripts from specific Nanopore protocols. In summary, LIQA allows users to get improved gene and isoform abundance estimates from long-read RNA-seq data.***

***The authors benchmarked the results of running LIQA on simulated and real datasets against those of established tools (FLAIR and Mandalorion) and showed that their tool particularly outperforms others when there is the need to correct for the aforementioned 3´ end bias.***

***We praise the authors for a well-structured manuscript, the clever usage of established mathematical models (such as analysing read length as censored data to apply Kaplan-Meier estimators in the quantification of truncated or incomplete reads), and developing an interesting tool that builds upon state-of-the-art approaches. The organisation and separation between methodological aspects in Methods and Materials is very intelligible, with the main text also enabling a non-expert reader to grasp the critical features of LIQA without having to initially go through the all methodological intricacies.***

***However, as detailed below, there are several shortcomings in the rigour with which results are presented and interpreted. These need to be addressed before we can consider this work sound enough for publication.***

We want to start by thanking the reviewer for these insightful comments. As you will see below, we have made extensive efforts to address the concerns, and we appreciate the reviewer’s comments which have helped us improve both the quality of the computational tool and the presentation of the manuscript itself.

***MAJOR CONCERNS:***

***1. Several of the plots supporting the results lack enough details for objective examination. For example, the bar plots summarising Spearman's coefficients and RMSE values (e.g., Figures 2 and 3, Supplementary Figures 3 to 6) should include the actual values (for instance, on top of the respective bar), allowing the reader to unbiasedly assess the magnitude of differences between tools.***

We apologize for the lack of details in these Figures. We have updated Figures 1-5 and most of Supplementary Figures with more detailed information. For example, following your suggestion, we have summarized and presented Spearman’s correlation and RMSE values in the bar plot for each method in Figure 2, 3, Supplementary Figure 3, 4 and 8. Moreover, for DAS analysis, we have presented AUC values, correlation values and FDR-values in Figures 3 and 5. Hopefully, these updated figures now clearly display our results to reviewers and readers.

***2. More importantly and related to the previous point, the results are generally described with a bias towards LIQA that is typically only supported by subjective examination of plots. For instance, the usage of "clearly" is too strong when results only slightly favour LIQA. We urge the authors to avoid subjective overstatements and empirically and/or statistically support their observations. For instance, when describing the results in figure 3(B), in lines 180-181, the authors claim that "LIQA appears to be much more accurate than the other two methods" while visual inspection suggests that the difference in Spearman's correlation between LIQA and FLAIR is roughly equal or smaller than that found for panel A, where FLAIR is actually more accurate than LIQA for shorter isoforms. Moreover, the significance of the reported differences in correlation should be statistically tested and the respective p-values should be included in the respective figures.***

We apologize for the bias description in the Results section. Following your suggestion, we have removed the subjective over-statements. For example, we have removed the strong word “clearly” in the manuscript. Also, we revised the summary sentence to “LIQA is more accurate than the other four methods at 5’ terminal exon, especially when sequencing depth is low.”

Following your suggestions, we considered two ways to statistically test the performance improvement of LIQA. First, we tested the difference between LIQA and other long-read methods based 10 simulation datasets. As shown in Supplementary Table 2, for relative abundance estimation, LIQA is more accurate than FLAIR and TALON with 9.7% (p-value=0.034) and 9.3% (p-value=0.021) higher Spearman’s correlation, respectively. These statistical test results indicate that LIQA significantly performs better than other approaches for genes with low read coverage. Second, we tested the difference between different model of LIQA as following:

Full model that accounts for both read length bias and read quality score:

$$L\left( \tilde{\boldsymbol{\Theta}} | \mathbf{R},\mathbf{Z} \right)=\prod_{r\in\boldsymbol{R}} \prod_{i\in\boldsymbol{I}} \left( q\left( r,i \right)\cdot f\left( L_{r} \right| iso. =i)\tilde{\theta}_{i} \right)^{\boldsymbol{Z}_{\boldsymbol{R},\boldsymbol{I}}\left( r,i \right)}$$

Read length model that accounts for read length bias but not read quality score:

$$L\left( \tilde{\boldsymbol{\Theta}} | \mathbf{R},\mathbf{Z} \right)=\prod_{r\in\boldsymbol{R}} \prod_{i\in\boldsymbol{I}} \left( f\left( L_{r} \right| iso. =i)\tilde{\theta}_{i} \right)^{\boldsymbol{Z}_{\boldsymbol{R},\boldsymbol{I}}\left( r,i \right)}$$

Read quality model that accounts for read quality score but not read length bias:

$$L\left( \tilde{\boldsymbol{\Theta}} | \mathbf{R},\mathbf{Z} \right)=\prod_{r\in\boldsymbol{R}} \prod_{i\in\boldsymbol{I}} \left( q\left( r,i \right)\cdot\tilde{\theta}_{i} \right)^{\boldsymbol{Z}_{\boldsymbol{R},\boldsymbol{I}}\left( r,i \right)}$$

Baseline model without bias correction:

$$L\left( \tilde{\boldsymbol{\Theta}} | \mathbf{R},\mathbf{Z} \right)=\prod_{r\in\boldsymbol{R}} \prod_{i\in\boldsymbol{I}} \left( \tilde{\theta}_{i} \right)^{\boldsymbol{Z}_{\boldsymbol{R},\boldsymbol{I}}\left( r,i \right)}$$

As shown in Supplementary Table 3, full model significantly performed better than baseline model with P-value less than 0.001 (df=189). These statistical test results are consistent with Figure 3(C)(D) and indicate the significant improvement for modeling read quality score and read length bias. We have revised the manuscript and described the results more accurately.

***3. In lines 240-242, it is claimed that the alleged better performance of LIQA is due to accounting for isoform length bias and base quality scores. Although plausible, this is never demonstrated. For instance, the differences in correlation of TPM between tools do not exhibit an obvious isoform length-associated trend. The actual performance of the benchmarked tools needs to be profiled against different levels of length bias and qualitatively related with base quality scores for the merits of accounting for those effects to be stated.***

Thanks very much for your constructive suggestions. We agree that it is important to assess the merits of accounting for read length bias and read quality.

First, we evaluated the impact of degree of non-uniformity in read coverage on isoform estimation. We considered the chi-square statistics that measures the goodness-of-fit of coverage uniformity. Isoforms were divided into two categories based on median of the corresponding measure (Chi-square statistic > median, Chi-square statistic < median). Isoform expression with non-uniform read coverage is more challenging to estimate because the 5’ end is less likely to be covered by sequencing reads compared to 3’ end. Figure 3(B) shows the comparison of Spearman’s correlation for five methods. For isoforms with length less non-uniform read coverage, Spearman’s correlations of LIQA and FLAIR are close. However, LIQA outperforms the other four methods for isoforms with more non-uniform read coverage (Chi-square statistic > median). The improvement of LIQA compared to FLAIR is 5% higher for these isoforms. We observed similar pattern when accuracy was quantified by RMSE values. This is likely because LIQA models potential truncated reads which result 3’ coverage bias when quantifying isoform expression.

Second, we have conducted a new study based on the simulation data using four different models of LIQA as following:

Full model that accounts for both read length bias and read quality score:

$$L\left( \tilde{\boldsymbol{\Theta}} | \mathbf{R},\mathbf{Z} \right)=\prod_{r\in\boldsymbol{R}} \prod_{i\in\boldsymbol{I}} \left( q\left( r,i \right)\cdot f\left( L_{r} \right| iso. =i)\tilde{\theta}_{i} \right)^{\boldsymbol{Z}_{\boldsymbol{R},\boldsymbol{I}}\left( r,i \right)}$$

Read length model that accounts for read length bias but not read quality score:

$$L\left( \tilde{\boldsymbol{\Theta}} | \mathbf{R},\mathbf{Z} \right)=\prod_{r\in\boldsymbol{R}} \prod_{i\in\boldsymbol{I}} \left( f\left( L_{r} \right| iso. =i)\tilde{\theta}_{i} \right)^{\boldsymbol{Z}_{\boldsymbol{R},\boldsymbol{I}}\left( r,i \right)}$$

Read quality model that accounts for read quality score but not read length bias:

$$L\left( \tilde{\boldsymbol{\Theta}} | \mathbf{R},\mathbf{Z} \right)=\prod_{r\in\boldsymbol{R}} \prod_{i\in\boldsymbol{I}} \left( q\left( r,i \right)\cdot\tilde{\theta}_{i} \right)^{\boldsymbol{Z}_{\boldsymbol{R},\boldsymbol{I}}\left( r,i \right)}$$

Baseline model without bias correction:

$$L\left( \tilde{\boldsymbol{\Theta}} | \mathbf{R},\mathbf{Z} \right)=\prod_{r\in\boldsymbol{R}} \prod_{i\in\boldsymbol{I}} \left( \tilde{\theta}_{i} \right)^{\boldsymbol{Z}_{\boldsymbol{R},\boldsymbol{I}}\left( r,i \right)}$$

Figure 3(C)(D) show the isoform estimation accuracy comparison results using different models. For isoforms with more non-uniform read coverage (Chi-square statistics > median), model with read length bias correction has 9% (Full model vs read quality model) and 10% (Read length model vs baseline model) higher Spearman’s correlation. For genes with less average read quality, model with read quality score has 6% higher Spearman’s correlation. Isoform estimation accuracy drops noticeably when using model with no read-length bias correction. We have revised the manuscript and added these evaluations in the Results section (page 9 paragraph 2).

***4. The results illustrated in Figure 5(E) are misinterpreted (lines 307-312). From the sashimi plots therein, one cannot conclude that the exon in the green square is higher in cancerous cells than in normal-like ESCC. Careful examination suggests a much lower coverage for the normal samples, particularly in the 5´ half of the transcript, clearly not enough to resolve that exon (read coverage in the vicinity is of only 3 reads, compared with >10 for the cancerous sample and dozens for the short-read data). The same lack of enough coverage to resolve DAS for the exon in the red box applies. In this example, there's no evidence for the alleged "flip-flop expression pattern" and, contrary to claimed by the authors (lines 310-312), the long-read data performs worse than short-read data, for which read coverage is much higher.***

We agree with the reviewer that the limited read coverage at this exon is not enough to show clear splicing difference between ESCC and normal cells. To make a fair comparison, we selected exons with higher coverage (>30) by both long-read and short-read data and made sashimi plot to examine empirical evidence for differential alternative splicing. In the revised manuscript, we illustrate another DAS exon from gene *CDV3* detected by LIQA but missed by PennDiff using short-read data. The differential alternative splicing expression of this gene between ESCC and normal cells was reported by previous study[13]. As shown in Figure 5(E), the sashimi plots of gene *CDV3* indicates that the relative expression of exon in the red square is lower in cancerous cells than normal-like ESCC. However, this event is missed by short-read data. The read coverage difference between normal-like and cancerous ESCC in sashimi plots indicates lesser usage of isoforms (NM_001134422, NM_0011134423, NM_001282765) which include this exon in the ESCC cells. We have revised the manuscript and added this more convincing example to Figure 5 and in the Results section.

***5. In the Discussion, the authors describe their concerns about finding the right tradeoff between robustness and running time, suggesting that future versions of LIQA may sacrifice the former to favour the latter. This reflection should be supported by precise information on the running time differences between the tools being compared. The running time of LIQA should be disclosed. How slower is LIQA than the other tools? Minutes? Hours? This is an important piece of benchmarking information. The decision of relaxing the robustness to get a faster analysis should ideally be tuned by the user, if sufficient information on this tradeoff is provided.***

Thanks for pointing this out. Following your suggestion, we have summarized the running time of different methods on simulation in Supplementary Table 1. (Following another reviewer’s suggestion, we have also added two additional computational approaches in comparative analysis, including the Oxford Nanopore Pipeline (ONP). ONP has the fastest runtime. ONP utilizes the alignment-free short-read approach Salmon, which requires less computational resource, and yields less accurate isoform expression estimates. LIQA is 30-40 minutes slower than other long-read approaches as it implements bias correction and EM algorithm. We have revised the manuscript and added this information in Discussion section. Hopefully, this information is now clear for the reviewer and for users.

***6. The whole DAS section is oversimplified, as only numbers of DAS events are compared, even though the precise profiling of such events is where the usage of long-read technology can prove advantageous. The precision of LIQA in quantifying the relative isoform abundance associated with DAS events (and not only the ability to detect them) should also be evaluated.***

We apologize for the lack of details on the results on DAS analysis. To evaluate the performance more comprehensively, we first generated ROC curve and precision-recall curve to compare the performance of LIQA with other methods in DAS detection. As shown in Figure 3(E), LIQA achieved AUC = 0.94 after FDR control (FDR < 0.05). Given FDR threshold equals to 0.05, LIQA gave the best performance with precision = 0.98 and recall = 0.78. Compared to LIQA, the second-best performer FLAIR yields 0.1%, 0.3%, 3.5% less in precision, recall and AUC respectively. Second, following your suggestion, we examined isoform relative abundance accuracy from correctly and incorrectly detected DAS genes. After FDR control, we identified 537 out of 2,465 genes to be significantly differentially spliced, of which 431 are true positives and 1836 are true negatives. For these correctly predicted genes, true isoform relative abundance is highly correlated with LIQA’s estimates (Spearman correlation= 0.91). For false positive and negative genes, the spearman correlation is 38% lower compared to true positive and negative. This is not surprising because accurate estimation of isoform expression level leverage the power of regression model in detecting DAS events. We have revised the manuscript and added these analysis results to Results section (page 9-10).

***MINOR ESSENTIAL REVISIONS:***

***7. LIQA should be more easily installable as a Python package via pip (instructions on how to do so in Python's official documentation available at***

***(***[***https://packaging.python.org/tutorials/packaging-projects***](https://packaging.python.org/tutorials/packaging-projects)***).***

Thanks for pointing this out. Following your suggestion, we have substantially improved the program and documentation for LIQA. Specifically, we have published LIQA to PyPi to simplify installation for users. Also, we have changed the isoform annotation input file format to GTF. Moreover, we are making Conda as well as docker image for LIQA. A few users already used LIQA and have communicated feedback to us or submitted GitHub issues to us. The usability of LIQA has been significantly improved now.

***8. Supplementary figures (except for Supplementary Figure 1) are not mentioned in the manuscript. We agree with the inclusion of those supplementary figures but they should be properly referenced across the manuscript.***

Thanks. We have revised the manuscript and added references for every figure in the supplementary material.

***9. In line 225-228, the authors mention that, when applying LIQA to universal human reference RNA-seq data, they decided to filter out from the qRT-PCR measurements genes with a single isoform "because estimating isoform-specific expression for these single-transcript genes is trivial". Although this may be true, a typical user will also be interested in these unique isoform genes and they would still serve as positive controls for LIQA's performance. They should therefore be included the comparison, marked as a separate group.***

Following your suggestion, we conducted additional simulations and real data analyses for genes with only one isoform. As all isoform relative abundances equal to 1, we measure the similarity between estimated TPM and the ground truth using Spearman’s correlation and RMSE. As shown in Supplementary Figure 4 and Supplementary Figure 8(A), all methods have similar performance in isoform usage quantification. This is not surprising because expression quantification of genes with single isoform is much easier than genes with 2 and more isoforms. All methods compute TPM based on gene-level read count without conducting read deconvolution process.

***10. In the Abstract, lines 31-32, sentence "Moreover, given isoform usage estimates, (...)" does not convey that LIQA is able to provide these values and may lead to confusion on whether the user needs to provide LIQA with such data.***

Thank you. We have rephrased the sentence to “Moreover, LIQA can detect DAS events between conditions using isoform usage estimates.”

***11. In the Abstract, lines 32-34, in sentence "We evaluated (...) and demonstrated that it [LIQA] outperforms other approaches in rare isoform characterization (...)", it is not clear 1) what "rare" means in this context and 2) how this was quantified, as no support for such observation can be found in the manuscript.***

We apologize for the lack of clarity. We have rephrased this sentence to “We evaluated LIQA’s performance on simulated data and demonstrated that it outperforms other approaches in characterizing isoforms with less read coverage and in detecting DAS events between two groups.” Simulation study results indicate that LIQA performs better than the other 4 approaches especially when sequencing depth is lower (for example, on the 0.5M simulation data set).

***12. In the Introduction, lines 53-55, in sentence "However, due to limited read length (ranges from 50 to 150 nucleotides) transcripts cannot be (...)" the presented length range definition of short reads is over-simplistic, given the intention of motivating the usage of long reads. Short reads can be 200nt-long and still lack the properties of longer reads. Moreover, providing an estimate for the range of mRNA transcripts can help illustrating the convenience of using long reads. This would also facilitate the interpretation of "allows a large portion or the entire mRNA or cDNA molecule to be sequenced" in line 72.***

Thanks for the suggestion. We summarized the isoform length distribution in the following table.

| Quantile | Min | 25% | 50% | Mean | 75% | Max |
| --- | --- | --- | --- | --- | --- | --- |
| Isoform length (bp) | 8 | 594 | 1,543 | 2,121 | 2,841 | 205,012 |

Also, we have rephrased this sentence to “However, due to limited read length, it is challenging to accurately characterize transcript using short reads as 81% of isoforms have length greater than 500bp under GENCODE annotation (median = 1,543 bp and mean = 2,121 bp).” to better illustrate the advantage of using long-read data for transcriptome studies.

***13. In the Introduction, lines 63-64, the fact that "different bias correction algorithms can result in conflicting estimates" does not mean that "(...) fragmented short reads cannot quantify isoform expression accurately (...)". Although results can be conflicting, some programs may accurately estimate isoform expression. Such bold statement would need support from rigorous benchmarking.***

Thanks for pointing this out. Instead of making this bold statement, we have rephrased this sentence to “Overall, quantifying isoform expression using fragmented short reads remains challenging, especially at complex gene loci.” Also, we cited Hu et al[4], Steijger et al[5] and Tilgner et al[6]’s work as references to support this statement.

***14. In the Introduction, lines 73-76, supporting references for the contribution of long-read RNA-seq to "rare isoform discovery, isoform expression quantification and DAS event detection" are missing and the mention to the possibility of detecting RNA modifications based on the "success of DNA modification detection" (also missing a reference) is speculative and does not positively contribute to the manuscript's flow.***

Thanks for pointing this out. We have cited Au et al.[8], Clark et al.[9], Sharon et al.[10], Gupta et al.[11] and Tilgner et al.,[12]’s work as reference to support this statement. We agree that RNA modification detection does not positively contribute to the manuscript’s flow and have removed this sentence from the manuscript.

***15. In the Introduction, lines 77-79, sentence "However, there are still a few unique challenges to analyze long-read RNA-seq data because existing methods developed for Illumina short-read RNA-seq do not have optimal performance when directly used on long-read RNA-seq." is lacking a reference. In the absence of a reference, an appropriate justification for the assumptions underlying the non-applicability of short-read RNA-seq methods to long reads is required.***

Thanks for pointing this out. For isoform expression quantification using long-read data, the methods should account for low read coverage and misalignment resulted from high sequencing error rate. However, parametric bias correction of short-read approaches require high read coverage to yield stable parameter estimation. For example, Wan et al.[14] proposed a parametric model that specifically models the non-uniformity caused by RNA degradation. Roberts et al.[15] developed a variable length Markov model that corrects both sequence and positional bias. Nicolae et al.[16] implemented a reweighting scheme to correct for hexamer and repeat bias. Moreover, Li et al.[17] proposed a quasi-multinomial model with a single parameter to capture positional, sequence and mapping biases.

Second, to account for high error rate of long reads, the isoform-read assignment model should be designed to have tolerance to small range of misalignment while short-read approaches require accurate alignment of reads at intron/exon boundary.

Following your suggestion, we have added the sentence “This is because that parametric bias correction of short-read approaches require high read coverage and isoform-read assignment is not robust to small range misalignment from long-read data.” to the Introduction section and cited Wan et al, Roberts et al., Nicolae et al., and Li et al.’s work as references. Hopefully this justification is now clearer to the reviewer and readers.

***16. In the Introduction, lines 100-101, sentence "Our results demonstrate that LIQA outperforms existing methods in isoform expression quantification." is a misleading overstatement, given that is not descriptive enough. LIQA does not outperform existing methods in all conditions.***

We apologize for the over-statement. We have changed this sentence to “Our results demonstrate that LIQA is an accurate approach for isoform expression quantification accounting for read coverage bias and high error rate of long-read data”. This sentence is a more accurate conclusion and consistent with our results.

***17. Although the manuscript's Figure 1 version is more appealing aesthetically, the directionality provided in GitHub's version of the same flowchart illustrating LIQA is more informative on how the program works. In the manuscript's Figure, the top and bottom boxes in panel A suggest two different levels of information/data types. Moreover, it is not clear what the input and output of each step of analysis are. For instance, the flowchart misleadingly suggests that LIQA aligns raw RNA-seq data.***

Thanks and we agree. Following your suggestion, we have updated the flowchart in Figure 1. This flowchart is more informative and has highlighted the input and output data at each step. Hopefully, this version is now clearer to the reviewers and readers.

***18. Results, line 107: it is not clear what "read length probability" refers to at this stage of the manuscript.***

Thanks for pointing this out. We have changed “read length probability” to “read coverage bias”.

***19. Figure 2 and Supplementary Figure 1 would be more comparable if the X-axes are kept the same across the plots. The numbers indicating the median read lengths and median count in each of the downsampling libraries should also be included in the plots in Supplementary Figure 1 (like in Figure 2A).***

Thanks for pointing this out. We have modified Supplementary Figure 1 following your suggestions. The range of X-axis are consistent. Median read length and median count have been highlighted.

***20. In Results, lines 143-145, the mention to "the coverage plots of the simulated data" should be accompanied by a reference to those plots, which are to be shown as Supplementary Figures.***

Thanks for the suggestion. We have generated the sashimi plot of the simulated data and added it to Supplementary Figure 1(B).

***21- It is not clear what distributions are represented in the boxplots for the DAS statistics (recall, precision and F1 score) in Figure 3C. Do these refer to 10 different simulations? If so, points corresponding to the 10 values should be part of the plot as well. Also, the line width used is in those plots is too thick for the reader to discriminate quartiles.***

Yes, boxplots in Supplementary Figure 5(B) show the distribution of DAS statistics (recall, precision and F1 score) from 10 different simulations. Following your suggestion, we have added corresponding points to the figure and changed the thickness of the boxplot.

***22. Figures 4(A) and 4(B) and their captions could be improved. What the red line in panel A is should be explicitly stated. The colours of dots in panel A should match those of the legend and of panel B. The colour legend should be closer to panel B. The Y-axis label is missing in panel B.***

Thanks for pointing this out. Black line in Figure 4(A) represents the local polynomial regression line fitted with estimates and qRT-PCR in log scale, which facilitates the examination of consistency between estimates and gold standard. We have added this description into the Figure caption. Also, we have changed the color dots in panel A to match each method. The Y-axis label is “correlation”. The X-axis labels are “Pearson correlation” and “Spearman correlation”. As X-axis labels contains the information of “correlation”, we omitted the Y-axis label.

***23. In Figure 5(E), "Long-read" and "Short-read" should be the actual titles of the two panels and not be misleadingly placed near the representation of the annotated isoforms.***

Thanks for pointing this out. Following your suggestion, we have placed “Long-read” and “Short-read” as actual titles.

***24. Regarding the Kaplan-Meier plots in Supplementary Figures 7 to 9, the authors should consider marking the isoform length in the plots to facilitate interpretation.***

Thanks. Following your suggestion, we have modified Supplementary Figure 9-11 by marking the isoform length for each transcript.

***25. Supplementary Figures 7 and 8 should have at least some genes in common, for comparison.
Although the first paragraph of Methods is written with clarity and we congratulate the authors for introducing the mathematics with intelligible explanations, the meaning of ZR,I (line 380) is not explained.***

Thanks for pointing this out. Following your suggestion, we selected out 10 genes in common and show their read length probability plot in Supplementary Figures 10, 11 to facilitate the comparison.

We apologize for the confusion of parameter $\boldsymbol{Z}_{\boldsymbol{R},\boldsymbol{I}}$. We have revised the description to “For read-isoform assignment, LIQA accounts for incorrect alignment at splice site. We define parameter $\boldsymbol{Z}_{\boldsymbol{R},\boldsymbol{I}}$ as a $|\boldsymbol{R}|\times|\boldsymbol{I}|$ a read-isoform compatibility matrix with $\boldsymbol{Z}_{\boldsymbol{R},\boldsymbol{I}}\left( r,i \right)=1$ if long-read $r$ is generated from a molecule that is originated from isoform $i$ (number of mismatch base pairs < 20 bp instead of exact match), and $\boldsymbol{Z}_{\boldsymbol{R},\boldsymbol{I}}\left( r,i \right)=0$ otherwise” in Method section. Hopefully this definition is now clearer to reviewers and readers.

***26. Methods, lines 407-408, "The read is treated as complete when the distance between its ending alignment position and any known isoform 5' end is less than 20 bp (Figure 1(B)).": the authors should explain the choice of a threshold of 20 bp and include this information in the figure scheme.***

Thanks for pointing this out. LIQA utilizes statistical survival model to account for 3’ sequence bias. Therefore, we only consider reads with ending alignment position at the 5’ end. Due to alignment and sequencing errors, ending positions of reads might not be consistent with annotated isoform boundary. To account for the small range alignment error, we treat a read as complete when the distance between its ending alignment position and any known isoform 5’ end is less than 20 bp in our analysis. We tested the sensitivity of LIQA to this threshold when estimating read length probability. 20 bp is a robust distance for read-isoform assignment and the affect read length probability curve is trivial. Moreover, we modified LIQA program to allow user to specify this threshold in analysis. We have added this information to Figure 1(A) and revised the manuscript accordingly.

***PROOFREADING EDITS:***

***27. Line 59: "tackle" instead of "tackle".***

Thank you. We have corrected this.

***28. Line 107: "corrects" instead of "correct".***

Thank you. We have corrected this.

***29. Line 114: "expression" instead of "expressions".***

Thank you. We have corrected this.

***30. Line 156: Figure 2(A) should be 2(B,C) and, similarly, Figure 2(B) in line 160 should be 2(C).***

Thank you. We have corrected this.

***31. Line 162: "higher" should be "lower", as this refers to the RMSE.***

Thank you. We have corrected this.

***32. Line 191: "we sampled relative abundances of isoforms" instead of "we sampled isoform relative abundances of isoforms".***

Thank you. We have corrected this.

***33. Line 201: "outperform" instead of "outperforms".***

Thank you. We have corrected this.

***34. Line 211: "hardly a full" instead of "hard to be full".***

Thank you. We have corrected this.

***35. Line 224: "gold" instead of "golden".***

Thank you. We have corrected this.

***36. Line 238: one "only" should be removed at the end of the sentence.***

Thank you. We have corrected this.

***37. Line 240: "major reason for the better performance of LIQA" instead of "major reason of the better performance for LIQA".***

Thank you. We have corrected this.

***38. Lines 245-248: "severe 5´ degradation" instead of "severe 3' degradation" and "Terminal exons at the 5´ end in the red square" instead of "Terminal exons at 3' end in red square.***

Thank you. We have corrected this.

***39. Line 255: "Acute myeloid leukemia (AML)" instead of "AML", as the acronym needs to be introduced.***

Thank you. We have corrected this.

***40. Line 257: URL*** [***https://denbi-nanopore-training-course.readthedocs.io/en/latest/basecalling/basecalling.html%23references***](https://denbi-nanopore-training-course.readthedocs.io/en/latest/basecalling/basecalling.html%23references) ***returns error "This page does not exist yet." and should be fixed.***

Thank you. We have updated the link to “<https://denbi-nanopore-training-course.readthedocs.io/en/latest/basecalling/basecalling.html>”

***41. Line 264: "analyze short-read sequencing data for the same AML sample" instead of "analyze a short-read data sequenced based on the same AML sample".***

Thank you. We have corrected this.

***42. Line 265: "reads" instead of "read".***

Thank you. We have corrected this.

***43. Line 271: "surprising" instead of "surprised".***

Thank you. We have corrected this.

***44. Line 288: "differences" instead of "difference".***

Thank you. We have corrected this.

***45. Line 296: "most frequent type of event" instead of "richest event".***

Thank you. We have corrected this.

***46. Line 298: "type" instead of "types".***

Thank you. We have corrected this.

***47. Line 325: "LIQA is more effective" instead of "LIQA is effective".***

Thank you. We have corrected this.

***48. Line 339: "files" instead of "file".***

Thank you. We have corrected this.

***49. Line 346: "read coverage" instead of "reads covered".***

Thank you. We have corrected this.

***50. Line 348: "may be" instead of "maybe".***

Thank you. We have corrected this.

***51. Line 354: "per-base" instead of "per-based".***

Thank you. We have corrected this.

***52. Line 411: "isoforms are treated" instead of "isoforms is treated".***

Thank you. We have corrected this.

***53. Line 425: "defined" instead of "deifned".***

Thank you. We have corrected this.

***54. Legends of Supplementary Figures 10 and 11: it must be stated what red and blue Sashimi plots correspond to.***

Thank you. We have corrected this.

**-------------------------------------------------------**

***Reviewer #3: The authors present a novel statistical method, LIQA, for isoform-level quantification and differential splicing detection from long read RNA-Seq data, such as that generated by Oxford Nanopore (ONT) and Pacific Biosciences (PacBio).  The novelty of LIQA's quantification approach relative to competing methods is that it attempts to model the uncertainty of the assignment of each long read to its isoform of origin.  Critical aspects of the statistical model include a model of read lengths that takes into account the phenomenon of long reads that are truncations of full transcripts and a model of the sequence similarity (referred to as the "quality score") between a long read and a candidate isoform.  LIQA performs quantification by finding maximum likelihood estimates of its statistical model, via the EM algorithm.

LIQA's performance is compared to two competing methods, FLAIR and Mandalorion, using both simulated data and real data from the well characterized Universal Human Reference (UHR) RNA sample.  Results indicate that LIQA generally performs better than the other two methods.  In addition, two case studies of using LIQA on data from AML and ESCC samples are presented.***

***In general, a more rigorous statistical treatment of isoform quantification from long read data is a welcome contribution.  And the breadth of data sets on the methodology is evaluated is excellent.  My major concerns with the current manuscript are with regard to the evaluation criteria used and lack of justifications for the components of the model used by LIQA.***

Thank you for the summary of our manuscript and for these insightful comments. Our detailed response is given below, and we believe the manuscript is substantially improved by addressing these comments.

***Major comments:***

***1. From what I can understand from the manuscript, each long read is mapped to one gene, but there may be ambiguity as to which isoform of the gene the long read is derived from.  Therefore, the only aspects of the quantification to be evaluated are the relative abundances of the isoforms within each gene.  The evaluations of "global" gene expression or isoform expression are not informative, or are at least redundant with the evaluation of the within-gene relative abundances.  I would recommend restricting evaluations to the within-gene relative abundances.  In addition, it does not seem proper to pool relative abundances from isoforms across all genes and compute correlations; the unit of evaluation is really at the gene level, since the within-gene relative abundances of the isoforms are highly correlated.  I would recommend computing an accuracy metric for each gene that represents how well the isoforms of that gene are ranked/quantified, and then summarizing these gene accuracies.***

Thanks for raising this question. Based on GENCODE annotation, 90.2% genes have less than 4 isoforms. For these genes, within-gene correlation metric estimation may be biased due to limited data points. To remove the high correlation within one gene, we considered two other ways to measure estimation accuracy at the gene-level. First, we used Hellinger distance to quantify the distance between underlying isoform relative abundance and estimates for each gene. Hellinger distance is a measure that describes the similarity between two probability distributions. Specifically, for gene with isoform relative abundance $\boldsymbol{\theta}=(\theta_{1},\ldots,\theta_{I})$ and $\hat{\boldsymbol{\theta}}=(\hat{\theta}_{1},\ldots,\hat{\theta}_{I})$, their Hellinger distance is defined as $H\left( \boldsymbol{\theta},\hat{\boldsymbol{\theta}} \right)=\frac{1}{\sqrt{2}}\sqrt{\sum_{i=1}^{I} \left( \sqrt{\theta_{i}}-\sqrt{\hat{\theta}_{i}} \right)^{2}}$. We have summarized the results in Supplementary Figure 4(B). Second, we summarized Spearman’s correlation of TPM and relative abundance for major-used isoform, which is the isoform with the highest relative abundance in each gene. As shown in Supplementary Figure 4(C), the estimation accuracy has been improved by ~5% as there are more read coverage for major isoforms. LIQA still yields more accurate isoform expression estimates with no within-gene isoform correlation bias.

***2. A number of aspects of the quantification model are unclear:***

***(A) why is the probability of a read originating from an isoform proportional to the product of the isoform length and the isoform abundance?  It is clear why the length factors into this for short-read RNA-seq data due to the fragmentation aspects of the protocol, but it is not clear why this is a factor in long-read (often full-length) RNA-seq data.***

We agree with the reviewer. We have revised the Methods section and LIQA program by assuming isoform relative abundance equals to probability of a read originating from the isoform. This modification only changes estimation of isoform relative abundance but does not affect TPM/RPG as they are based on $P\left( iso.=i \right)$ directly. We updated Figure 2(C) which shows comparison between methods in isoform relative abundance estimation. Compared to previous results, Spearman’s correlation and RMSE of LIQA are improved by 3.5% and 3.8%, respectively.

***(B) It does not appear that the read quality scores take into account indels.  Is that the case?  If so, this seems odd given how common indels are in long-read data, especially ONT.***

Thanks for pointing this out. We have modified the LIQA program and provided an option for user to determine whether to model read bases mapped to exonic region only or all bases from the reads. Moreover, we conducted additional analysis of 5M simulation data by comparing these two models. The difference of evaluation metrics, Spearman correlation and RMSE, are both less than 0.5% ($\Delta$Spearman Correlation = 0.33%, $\Delta$RMSE = 0.19%).

***(C) The generative model for the read length is a bit odd, in that it is defined via Bayes rule and it seems to be constructed by assuming that we draw from a read length distribution first, and then select among the isoforms that are larger than that read length.  That seems counter to the generative process of the sequencer, which would first select the transcript and then generates the read given that transcript.***

Thanks for pointing this out. The motivation of estimating read length distribution is to account for pore blocking and fragmentation during library preparation of ONT RNA sequencing protocol. Ideally, we can use informative reads with known isoform origin to estimate isoform-specific read length distribution. However, this is very challenging because these informative reads are sparse especially for long-read RNA-seq data. This may bias the isoform-specific read length distribution estimation due to limited sample size. Therefore, we estimate read distribution first using all reads from the gene, then apply Bayesian formula to approximate isoform-specific read length distribution. This strategy is a trade-off between bias and variance. This estimator is much more stable using all reads from the gene and allows us to assign different weights to read with different lengths in isoform quantification process.

***(D) Are reads only allowed to be truncated at the 5' end, or are 3' truncations allowed as well?***

Thanks. To reduce the number of parameters in the model, we only consider reads truncated at the 5’ end to model 3’ coverage bias when quantifying isoform expression. Otherwise the model might be unidentifiable due to limited read count data.

***3. In the evaluation of DAS, it is unclear how the predictions for the various methods were thresholded, i.e., was a FDR of 0.05 targeted?  It would be more informative to show a precision-recall curve here with the point obtained with the given threshold highlighted.***

To address this comment, we have included more summary statistics and figures in the Results section. To compare the performance of LIQA with other methods in DAS detection, we first generated ROC curve and precision-recall curve. As shown in Figure 3(E), LIQA achieved AUC = 0.94 after FDR control (FDR < 0.05). Given 0.05 FDR threshold, LIQA gave the best performance with precision = 0.98 and recall = 0.78. Compared to LIQA, the second-best performer FLAIR yields 0.1%, 0.3%, 3.5% less in precision, recall and AUC, respectively. Second, we examined isoform relative abundance accuracy from correctly and incorrectly detected DAS genes by LIQA. After FDR control, we identified 537 out of 2,465 genes to show DAS, of which 431 are true positives and 1,836 are true negatives. For these correctly predicted genes, true isoform relative abundance is highly correlated with LIQA’s estimates (Spearman’s correlation= 0.91). For false positive and negative genes, the spearman correlation is 38% lower than true positive and negative genes. This is not surprising because accurate estimation of isoform expression level leverages the power of regression model in detecting DAS events. We have revised the manuscript and added these analysis results to Results section (page 10).

***4. It is unclear how the DAS methodology differs from that of the other methods.  More discussion of this is warranted.  In addition, could the DAS methodology presented here be applied to the outputs of the other quantification methods, or is it specific to the output of LIQA?***

We apologize for the lack of clarity on the difference between LIQA and other methods in detecting differential alternative splicing (DAS) event. LIQA utilizes Gaussian coupla regression model to detect DAS. The main motivation of using Gaussian copula is to have a flexible framework to account for correlations of isoform relative abundance and to accommodate the fact that relative abundance ranges from 0 to 1 and follows a Beta distribution. Given the complexity of gene structure and the large number of isoforms for most mammalian genes, the correlation structure is often very complicated. To make valid statistical inference for downstream analysis, these correlations need to be taken into account. Since we do not know the joint distribution of the exon-inclusion levels, an alternative strategy is to model the correlation and the marginal distributions separately. Gaussian copula regression model is a commonly used statistical approach to tackle this type of problem. For isoform relative abundance level, we model its marginal distribution, which is assumed to be Beta distributed, using a generalized linear model. Then, a multivariate normal distribution is employed to link these marginal generalized linear models together to account for their correlations. In addition, isoform relative abundance estimates from other quantification methods can be applied to LIQA.

***Minor comments:***

***5. Figure 1A - I find this diagram to be confusing.  It would be clearer if there could be two types of objects in this diagram, processes (algorithms) and inputs/outputs (data), with the inputs and outputs of each process clearly shown and linked together.***

Thanks and we agree. Following your suggestion, we have updated the flowchart in Figure 1. This flowchart is more informative and has highlighted the input and output data at each step. Hopefully, this version is now clearer to the reviewer and readers.

***6. "Second, we compared the accuracy statistics for 5' terminal exon and 3' terminal exon of each isoform (Figure 3(B))"  What is being measured at these exons?***

We apologize for the lack of clarity on this. Isoform-level TPM estimates allow us to quantify global expression for each exon by summing up the expression levels of isoform which includes the exon. Based this relationship, we quantified global exon expression (TPM) of terminal exons to evaluate the impact of 3’ bias on estimation accuracy across different methods in Figure 3.

***7. "estimation accuracy of all methods is lower than simulated data because the qRT-PCR measures may not be accurate,": I'm not sure it is fair to directly fault the qRT-PCR measurements here; estimation accuracy could easily be worse simply because this is real data.***

Thanks for pointing this out. We have changed this sentence to “the estimation accuracy of all methods is lower than simulated data, especially for those transcripts with qRT-PCR measures close to 0.”

Reference

1.	Pan, Q., et al., Deep surveying of alternative splicing complexity in the human transcriptome by high-throughput sequencing. Nat Genet, 2008. 40(12): p. 1413-5.

2.	Wang, E.T., et al., Alternative isoform regulation in human tissue transcriptomes. Nature, 2008. 456(7221): p. 470-6.

3.	Wang, Z., M. Gerstein, and M. Snyder, RNA-Seq: a revolutionary tool for transcriptomics. Nat Rev Genet, 2009. 10(1): p. 57-63.

4.	Hu, Y., et al., PennSeq: accurate isoform-specific gene expression quantification in RNA-Seq by modeling non-uniform read distribution. Nucleic Acids Res, 2014. 42(3): p. e20.

5.	Steijger, T., et al., Assessment of transcript reconstruction methods for RNA-seq. Nat Methods, 2013. 10(12): p. 1177-84.

6.	Tilgner, H., et al., Defining a personal, allele-specific, and single-molecule long-read transcriptome. Proc Natl Acad Sci U S A, 2014. 111(27): p. 9869-74.

7.	Hafezqorani, S., et al., Trans-NanoSim characterizes and simulates nanopore RNA-sequencing data. Gigascience, 2020. 9(6).

8.	Au, K.F., et al., Characterization of the human ESC transcriptome by hybrid sequencing. Proc Natl Acad Sci U S A, 2013. 110(50): p. E4821-30.

9.	Clark, M.B., et al., Long-read sequencing reveals the complex splicing profile of the psychiatric risk gene CACNA1C in human brain. Mol Psychiatry, 2020. 25(1): p. 37-47.

10.	Sharon, D., et al., A single-molecule long-read survey of the human transcriptome. Nat Biotechnol, 2013. 31(11): p. 1009-14.

11.	Gupta, I., et al., Single-cell isoform RNA sequencing characterizes isoforms in thousands of cerebellar cells. Nat Biotechnol, 2018.

12.	Tilgner, H., et al., Comprehensive transcriptome analysis using synthetic long-read sequencing reveals molecular co-association of distant splicing events. Nat Biotechnol, 2015. 33(7): p. 736-42.

13.	Xie, Z.C., et al., Prognostic alternative splicing signatures and underlying regulatory network in esophageal carcinoma. Am J Transl Res, 2019. 11(7): p. 4010-4028.

14.	Wan, L., et al., Modeling RNA degradation for RNA-Seq with applications. Biostatistics, 2012. 13(4): p. 734-47.

15.	Roberts, A., et al., Improving RNA-Seq expression estimates by correcting for fragment bias. Genome Biol, 2011. 12(3): p. R22.

16.	Nicolae, M., et al., Estimation of alternative splicing isoform frequencies from RNA-Seq data. Algorithms Mol Biol, 2011. 6(1): p. 9.

17.	Li, W. and T. Jiang, Transcriptome assembly and isoform expression level estimation from biased RNA-Seq reads. Bioinformatics, 2012. 28(22): p. 2914-21.

**Second round of review**

**Reviewer 1**

Thank you to the authors for making big improvements on the manuscript, the comments are generally quite well addressed. I have some small comments left and hope the authors can reply:

For the UHR dataset (Figure 4), method performance differed a lot from simulated dataset. It may potentially be explained by the sequencing depth, but it would also raise a concern for the robustness of the method comparison conclusions. If possible, would probably be good to see when trying to increase the sequencing depth of UHR datasets, would similar conclusions from simulated datasets be observed for real datasets?

For the evaluation of read length bias correction impact, authors used the chi-square measurements to measure the coverage uniformity, however, how the chi-square measurements are derived is not described. It would be good if the authors could elaborate on this so that readers can understand and interpret it.

Typos:

Figure 3(C), “...chi-square statistic less than median…”, shouldn’t it be more than?

Presentations:

Figure 3(F) is a bit difficult to understand, there quite a lot overlapping in true positive/negatives vs false positive/negatives, would suggest to plot separately

**Reviewer 2**

We congratulate the authors on a much improved and more complete version of the manuscript. Most of our concerns were carefully addressed by the authors, with thorough explanations and appropriate edits to the text and figures. There are, however, specific details and clarifications missing (below) that the authors need to provide before we consider the manuscript ready for publication.

1. We generally agree with the alterations to phrasing proposed by the authors to address our major concern 2. However, the authors' response and Supplementary Tables 2 and 3 refer to "statistical test/comparison" without explicitly stating which test is performed and, importantly, what are its underlying assumptions. Details on the chosen tests need to be added for intelligibility and reproducibility. Along the same lines, differences in Spearman correlation values used, for instance, to claim that "LIQA is more accurate than the other four methods at 5' terminal exon" (and all statements associated with values in Figure 3, Supplementary Figure 3, etc.) must be statistically tested for significance and therefore accompanied by p-values (and an indication of the test performed). Similarly, when addressing our essential revision 9, the authors claim that, "as shown in Supplementary Figure 4 and Supplementary Figure 8(A), all methods have similar performance in isoform usage quantification". What makes the differences therein small enough for the performance to be considered "similar" and differences in other plots large enough to be relevant? Information on sequencing depth associated with the 20 simulated sets in Supplementary Table 2 must also be provided. We insist that that this manuscript cannot be deemed as acceptable for publication until the authors provide all the details necessary for full replication of their analyses and all statements involving quantitative differences are statistically supported.

2. The description of the analyses of read coverage uniformity can be made more intelligible to non-expert readers. For instance, the meaning of the two groups "chi-square > median" and "chi-square < median" and how these relate with isoforms showing more (or less) coverage uniformity are not properly introduced. Along the same lines, the left and right panels of Figure 3B could be labeled more intelligibly, with something like "more/less (non-uniform). The non-expert reader may also wonder why the authors talk about "less non-uniform" instead of "more uniform" (although we agree the former is statistically the correct phrasing), so perhaps some details and clarification can be included in the Methods.

3. We praise the authors for addressing our concern about Figure 5E with a new example exhibiting stronger supporting evidence for the advantage of using LIQA. However, the low resolution and small font sizes impair reading the number of reads associated with each junction in the sashimi plots.

4. We praise the authors for the useful comparisons provided in Supplementary Figures 10 and 11. However, the main purpose our original suggestion of "comparing at least some genes in common" between figures (essential revision 25) was the comparison between simulated and real isoform expression to address if major isoforms' read length probability estimations are similar. We therefore request the inclusion of genes in common between Supplementary Figures 9 (simulated) and 10/11 (real).

**Reviewer 3**

The authors have satisfactorily addressed my prior comments. However, a detail that has been revealed in the latest manuscript and in the response to reviewers is a bit concerning. Specifically, it appears that the gene annotation used for this study was GENCODE v24, which was released almost *six* years ago. The number of annotated isoforms in GENCODE has grown almost three-fold since that release. By my count, I see 60,651 genes and 234485 isoforms annotated in the latest (v37) GENCODE annotation. In this annotation, 39.4% of genes have more than one isoform, and these genes account for 84% of all isoforms. And 27% of genes have more than 4 isoforms.

My concern is that methods for quantifying isoforms can be severely impacted by the complexity of the annotated transcript set, and some may be more susceptible than others. Ideally, all of these analyses would have been done with a recent annotation. I understand that it may be a lot of work to redo everything with GENCODE v37, but perhaps the authors could provide some small additional experiments or evidence to show that with a more complex annotation, like GENCODE v37, that LIQA's performance remains acceptable and competitive.

**Authors Response**

**Point-by-point responses to the reviewers’ comments:**

***Reviewer #1: Thank you to the authors for making big improvements on the manuscript, the comments are generally quite well addressed. I have some small comments left and hope the authors can reply:***

***For the UHR dataset (Figure 4), method performance differed a lot from simulated dataset. It may potentially be explained by the sequencing depth, but it would also raise a concern for the robustness of the method comparison conclusions. If possible, would probably be good to see when trying to increase the sequencing depth of UHR datasets, would similar conclusions from simulated datasets be observed for real datasets?***

Thank you very much for the comments on the revised version of the manuscript. While we were unable to perform additional Nanopore direct mRNA sequencing on the UHR sample within a short period of time, we were able to download another PacBio-based long-read data on UHR with much higher coverage (5.6 million reads)[1]. We examined the intermediate file “counts_matrix_rpg.tsv” of FLAIR based on this dataset and found that the proportion of isoforms which have reads less than 3 dropped from 65% to 22% compared to our UHR dataset by direct mRNA sequencing (0.5 million reads). Next, we performed additional analysis to benchmark the isoform quantification accuracy using all approaches. As shown in Supplementary Figure 8(E), Pearson and Spearman’s correlations for each method are generally improved with increasing sequencing depth in the PacBio-based UHR dataset, compared to our UHR dataset by direct mRNA sequencing (Figure 4(B)). For example, Spearman’s correlation of FLAIR increased by 0.28 (from 0.45 to 0.73), whereas the corresponding values of LIQA is increased by 0.11. Nevertheless, LIQA still has the best performance among all methods. Based on this real dataset with increased sequencing depth, we further confirmed that LIQA is more robust to low read coverage compared to FLAIR, which performs well under relatively high sequencing depth. This conclusion from these two real UHR datasets is consistent with our analysis of simulation data with different sequencing depths (0.5M, 1M, 3M, 5M). We have revised the manuscript and added this description to the Results section.

***For the evaluation of read length bias correction impact, authors used the chi-square measurements to measure the coverage uniformity, however, how the chi-square measurements are derived is not described. It would be good if the authors could elaborate on this so that readers can understand and interpret it.***

We apologize for the lack of clarity on the chi-squared statistics to measure the coverage uniformity. Specifically, given an isoform of interest, we let $l$ denote the length and $O_{i}$ denote observed read coverage count at base pair position $i$. Total sequencing depth of this isoform $S=\sum_{1\leq i\leq l} O_{i}$. Under the uniform read coverage assumption, the expected read coverage count at each base pair position $E_{i}=S/l$. We apply Chi-squared goodness of fit statistics to measure the difference between observed read coverage and uniform read distribution. The test statistics is

$$\chi^{2}=\sum_{1\leq i\leq l} \frac{{(O_{i}-E_{i})}^{2}}{E_{i}}$$

The higher value of $\chi^{2}$ indicates that observed read coverage deviates more from uniform read distribution. We calculated $\chi^{2}$ for each isoform, then divided them into two categories based on median of the corresponding measure (more non-uniform: $\chi^{2}$ > median, less non-uniform $\chi^{2}$ < median) to evaluate the impact of read coverage distribution on isoform expression quantification. We have revised the manuscript and added this explanation of chi-squared statistics in the Methods and material section, and hopefully this explanation is now clearer to the reviewer and readers.

***Typos:
Figure 3(C), “...chi-square statistic less than median…”, shouldn’t it be more than?***

Thank you. We have corrected this typo.

***Presentations:
Figure 3(F) is a bit difficult to understand, there quite a lot overlapping in true positive/negatives vs false positive/negatives, would suggest to plot separately***

Thank you. Following your suggestion, we have plotted true positive/negative and false positive/negative separately in Figure 3(F).

-----------------------------------------------------

***Reviewer #2: We congratulate the authors on a much improved and more complete version of the manuscript. Most of our concerns were carefully addressed by the authors, with thorough explanations and appropriate edits to the text and figures. There are, however, specific details and clarifications missing (below) that the authors need to provide before we consider the manuscript ready for publication.***

Thank you very much for the positive comments on the revised manuscript. We have now addressed additional comments and please find our point-by-point response below.

***1. We generally agree with the alterations to phrasing proposed by the authors to address our major concern 2. However, the authors' response and Supplementary Tables 2 and 3 refer to "statistical test/comparison" without explicitly stating which test is performed and, importantly, what are its underlying assumptions. Details on the chosen tests need to be added for intelligibility and reproducibility. Along the same lines, differences in Spearman correlation values used, for instance, to claim that “LIQA is more accurate than the other four methods at 5’ terminal exon” (and all statements associated with values in Figure 3, Supplementary Figure 3, etc.) must be statistically tested for significance and therefore accompanied by p-values (and an indication of the test performed). Similarly, when addressing our essential revision 9, the authors claim that, “as shown in Supplementary Figure 4 and Supplementary Figure 8(A), all methods have similar performance in isoform usage quantification”. What makes the differences therein small enough for the performance to be considered “similar” and differences in other plots large enough to be relevant? Information on sequencing depth associated with the 20 simulated sets in Supplementary Table 2 must also be provided. We insist that that this manuscript cannot be deemed as acceptable for publication until the authors provide all the details necessary for full replication of their analyses and all statements involving quantitative differences are statistically supported.***

We apologize for the lack of details on the statistical tests and simulation data. We simulated ONT RNA-seq data 20 times to assess the statistical significance when comparing the performance of different methods. Each dataset includes 5 million (5M) reads. We also down-sampled 3 million (3M), 1 million (1M) and 0.5 million (0.5M) reads for the simulated data to evaluate the impact of sequencing depth on performance improvement of LIQA. We ran all methods with the same set of simulated aligned data in BAM format as input and calculated the Spearman’s correlation of TPM and relative abundance between true and estimated values. Based this metric from 20 simulated datasets, we conducted pairwise comparison of performance difference between all methods using paired Z-test. Mean difference, standard deviations, test statistics and P-values were calculated to support the statements in the manuscripts. Moreover, we conducted likelihood ratio test to compare different models of LIQA (full model, read length model, read quality model). Likelihood ratio test statistic $Q=-2(logL_{B}-logL_{A})$, where $L$ is the optimized likelihood function based on different models. All supplementary tables have been cited in the manuscript. For example, Supplementary Table 2 supports the statement “LIQA, FLAIR and TALON have higher Spearman’s correlation than Mandalorion and ONP for simulated datasets with low sequencing depth (1M, 0.5M)” in line 163 and “For simulated data with high sequencing depth (3M, 5M), LIQA has similar estimation accuracy with FLAIR and TALON based on Spearman’s correlation” in line 165. Supplementary Table 3 supports the statement “Comparison results at gene level reveal similar pattern (Supplementary Figure 4)” in line 169. Supplementary Table 4 supports the statement “LIQA is more accurate than the other four methods at 5’ terminal exon, especially when sequencing depth is low” in line 196 and statement “LIQA is more accurate than the other four methods for isoforms with less uniform read coverage (Chi-square statistic > median)” in line 206. Supplementary Table 7 supports the statement “Overall, isoform estimation accuracy drops noticeably when using baseline model (Supplementary Table 7)” in line 217. We have revised the manuscript and added the description of statistical tests in the Methods and material section and hopefully this explanation is now clearer to the reviewer and readers.

***2. The description of the analyses of read coverage uniformity can be made more intelligible to non-expert readers. For instance, the meaning of the two groups "chi-square > median" and "chi-square < median" and how these relate with isoforms showing more (or less) coverage uniformity are not properly introduced. Along the same lines, the left and right panels of Figure 3B could be labeled more intelligibly, with something like “more/less (non-uniform). The non-expert reader may also wonder why the authors talk about “less non-uniform” instead of “more uniform” (although we agree the former is statistically the correct phrasing), so perhaps some details and clarification can be included in the Methods.***

The other reviewer also raised the same concern and we apologize for the lack of clarity on the chi-squared statistics to measure the coverage uniformity. Specifically, given an isoform of interest, we let $l$ denote the length and $O_{i}$ denote observed read coverage count at base pair position $i$. Total sequencing depth of this isoform $S=\sum_{1\leq i\leq l} O_{i}$. Under the uniform read coverage assumption, the expected read coverage count at each base pair position $E_{i}=S/l$. We apply Chi-squared goodness of fit statistics to measure the difference between observed read coverage and uniform read distribution. The test statistics is

$$\chi^{2}=\sum_{1\leq i\leq l} \frac{{(O_{i}-E_{i})}^{2}}{E_{i}}$$

The higher value of $\chi^{2}$ indicates that observed read coverage deviates more from uniform read distribution. We calculated $\chi^{2}$ for each isoform, then divided them into two categories based on median of the corresponding measure (less uniform: $\chi^{2}$ > median, more uniform $\chi^{2}$ < median) to evaluate the impact of read coverage distribution on isoform expression quantification. We have revised the manuscript and added these explanations of chi-squared statistics in the Methods and material section.

Moreover, following your suggestions, we have changed the labels in Figure 3(B) to “More uniform” and “Less uniform”. Also, we replaced “less non-uniform” with “more uniform” and “more non-uniform” with “less uniform” to make the description of the analysis easier to understand for non-expert readers. Hopefully this interpretation and explanation are now clearer to the reviewers and readers.

***3. We praise the authors for addressing our concern about Figure 5E with a new example exhibiting stronger supporting evidence for the advantage of using LIQA. However, the low resolution and small font sizes impair reading the number of reads associated with each junction in the sashimi plots.***

We apologize for the low resolution and small font sizes in the sashimi plot. Following your suggestion, we have improved the figure resolution and splitted Figure 5(A-C) as Figure 5 and Figure 5(D-E) as Figure 6. Number of reads associated with each junction are now much clearer.

***4. We praise the authors for the useful comparisons provided in Supplementary Figures 10 and 11. However, the main purpose our original suggestion of "comparing at least some genes in common" between figures (essential revision 25) was the comparison between simulated and real isoform expression to address if major isoforms' read length probability estimations are similar. We therefore request the inclusion of genes in common between Supplementary Figures 9 (simulated) and 10/11 (real).***

Thanks for pointing this out. Following your suggestion, we selected 10 genes in common and showed their read length probability plot in Supplementary Figures 9, 10 and 11 to facilitate the comparison between simulated and real data.

***------------------------------------------------***

***Reviewer #3: The authors have satisfactorily addressed my prior comments. However, a detail that has been revealed in the latest manuscript and in the response to reviewers is a bit concerning. Specifically, it appears that the gene annotation used for this study was GENCODE v24, which was released almost *six* years ago. The number of annotated isoforms in GENCODE has grown almost three-fold since that release. By my count, I see 60,651 genes and 234485 isoforms annotated in the latest (v37) GENCODE annotation. In this annotation, 39.4% of genes have more than one isoform, and these genes account for 84% of all isoforms. And 27% of genes have more than 4 isoforms.***

***My concern is that methods for quantifying isoforms can be severely impacted by the complexity of the annotated transcript set, and some may be more susceptible than others. Ideally, all of these analyses would have been done with a recent annotation. I understand that it may be a lot of work to redo everything with GENCODE v37, but perhaps the authors could provide some small additional experiments or evidence to show that with a more complex annotation, like GENCODE v37, that LIQA's performance remains acceptable and competitive.***

Thanks very much for your constructive suggestions. We agree that it is important to assess the robustness of LIQA to a more complex annotation of isoforms. Following your suggestion, we have conducted a new simulation study based on the GENCODE v37, and generated 5 million Nanopore reads using NanoSim. We compared the performance of isoform expression quantification between LIQA and other methods. We summarized Spearman’s correlation of relative abundance for major-used isoform, which is the isoform with the highest relative abundance in each gene, and all isoforms. As shown in Supplementary Figure 4(D), LIQA still yields more accurate isoform expression estimates especially for major-used isoform with 7% higher spearman correlation than the second best performing method FLAIR.

Although not directly requested by the reviewers, during the revision process, we decided to evaluate the robustness of LIQA to over-annotation and under-annotation of isoforms. For over-annotation, we simulated RNA-seq reads based on 66% of the GENCODE v37 annotation. We then analyzed the simulated data with various methods using 100% of the GENCODE annotation, corresponding to 50% more of the true annotation. Supplementary Figure 4(E) shows the Spearman correlation results under different degrees of over-annotation. We find that the quantification accuracy of LIQA remained nearly unchanged as the degree of over-annotation increased. For under-annotation, we simulated RNA-seq reads based on 100% of the GENCODE v37 annotation. We then analyzed the simulated data with various methods using 50% of the GENCODE annotation, corresponding to 50% less of the true annotation. Supplementary Figure 4(E) shows the Spearman’s correlation results under different degrees of under-annotation. The estimation accuracy is 10% lower when 50% less of the true annotation was used in the analysis. Overall, LIQA is robust to over-annotation of isoform. For under-annotation, LIQA still yields more accurate results than other approaches.

1.	*Sequel II System Data Release: Universal Human Reference (UHR) Iso Seq*. 2020; Available from: <https://github.com/PacificBiosciences/DevNet/wiki/Sequel-II-System-Data-Release:-Universal-Human-Reference-(UHR)-Iso-Seq>.

**Third round of review**

**Reviewer 1**

The authors have addressed the remaining points. Figure 3F is still very difficult to read as symbols with identical colours are overlapping and I would suggest to change this.

**Reviewer 2**

We praise the authors for carefully and satisfactorily addressing most of our concerns. In particular, we thank them for a very thorough explanation on the statistical support of results, based on the issues we had raised. However, there are still a few details that require further clarification:

1. Please confirm and make explicit that all mean differences in Supplementary Tables 2 to 6, reflecting the paired Z-tests between pairs of tools, are supposed to be positive. If you are using absolute mean differences, please indicate so in the legend.

2. Unlike you state with “LIQA, FLAIR and TALON have higher Spearman’s correlation than Mandalorion and ONP for simulated datasets with low sequencing depth (1M, 0.5M)”, based on Supplementary Table 2, Mandalorion vs. TALON mean differences are not significant for any read coverage.

3. What is the support for the statement “For simulated data with high sequencing depth (3M, 5M), LIQA has similar estimation accuracy with FLAIR and TALON based on Spearman’s correlation”? Is this based on the non-significant p-values when comparing LIQA to FLAIR and TALON? A p-value > 0.05 does not prove the null hypothesis that the distributions are equal. Absence of evidence is not evidence of absence (of differences, in this case). Statistical tests can only be used to reject the null hypotheses, not to validate the. Anyway, that statement does not apply to the comparison between LIQA and TALON for 3M.

4. Finally, as a minor note for the sake of consistency, there are cells in these tables highlighted in red that do not have a p-value < 0.05 (e.g., Supplementary Table 2: FLAIR column, 1M, comparison to ONP shows a p-value of 0.081 that should not be highlighted in red).

**Reviewer 3**

The authors have fully addressed my prior comments. I have two minor comments on the revision:

1. Supp Fig 4 caption: for (D) it says "gene-level" TPM. I believe this should be isoform-level. If not, then I think the evaluation should be changed to isoform-level.

2. The chi-square statistic, as described, would seem to scale with the length of the isoform. I worry that instead of measuring uniformity, this is instead reflecting the length. When you do an actual Chi-square *test*, you take into account the degrees of freedom, which is length - 1 in this case.

**Authors Response**

**Point-by-point responses to the reviewers’ comments:**

***Reviewer #1: The authors have addressed the remaining points. Figure 3F is still very difficult to read as symbols with identical colours are overlapping and I would suggest to change this.***

Thanks for pointing this out. We have modified Fig. 3F by using different colors to mark true and false cases. With this change, the figure is now clearer to the reviewer and readers.

***Reviewer #2: We praise the authors for carefully and satisfactorily addressing most of our concerns. In particular, we thank them for a very thorough explanation on the statistical support of results, based on the issues we had raised. However, there are still a few details that require further clarification:***

***1. Please confirm and make explicit that all mean differences in Supplementary Tables 2 to 6, reflecting the paired Z-tests between pairs of tools, are supposed to be positive. If you are using absolute mean differences, please indicate so in the legend.***

We apologize for the lack of clarity on absolute mean differences. We have changed “mean difference” to “absolute mean difference” in the legend.

***2. Unlike you state with "LIQA, FLAIR and TALON have higher Spearman's correlation than Mandalorion and ONP for simulated datasets with low sequencing depth (1M, 0.5M)", based on Supplementary Table 2, Mandalorion vs. TALON mean differences are not significant for any read coverage.***

Thanks for pointing this out. To address this comment, we have changed the sentence to “LIQA has higher Spearman’s correlation than other methods for simulated datasets with low sequencing depth (0.5M) (Supplementary Table 2).”

***3. What is the support for the statement "For simulated data with high sequencing depth (3M, 5M), LIQA has similar estimation accuracy with FLAIR and TALON based on Spearman's correlation"? Is this based on the non-significant p-values when comparing LIQA to FLAIR and TALON? A p-value > 0.05 does not prove the null hypothesis that the distributions are equal. Absence of evidence is not evidence of absence (of differences, in this case). Statistical tests can only be used to reject the null hypotheses, not to validate the. Anyway, that statement does not apply to the comparison between LIQA and TALON for 3M.***

Thanks for pointing this out. To address this comment, we have changed the sentence to “For simulated data with high sequencing depth (3M, 5M), the Spearman’s correlation differences between LIQA, FLAIR and TALON are not significant (Supplementary Table 2).”

***4. Finally, as a minor note for the sake of consistency, there are cells in these tables highlighted in red that do not have a p-value < 0.05 (e.g., Supplementary Table 2: FLAIR column, 1M, comparison to ONP shows a p-value of 0.081 that should not be highlighted in red).***

Thank you. We have corrected this typo.

***Reviewer #3: The authors have fully addressed my prior comments. I have two minor comments on the revision:***

***1. Supp Fig 4 caption: for (D) it says "gene-level" TPM. I believe this should be isoform-level. If not, then I think the evaluation should be changed to isoform-level.***

Thank you. We have corrected this typo.

***2. The chi-square statistic, as described, would seem to scale with the length of the isoform. I worry that instead of measuring uniformity, this is instead reflecting the length. When you do an actual Chi-square *test*, you take into account the degrees of freedom, which is length - 1 in this case.***

We apologize for the lack of clarity on the test. We indeed used length-1 degree to account for different isoform lengths and have now added this description into Methods section. This description is now clear to readers.
